# Characterization of resistance to bacterial panicle blight in rice revealed by transcriptome and QTL mapping analyses

**DOI:** 10.3389/fpls.2026.1857873

**Published:** 2026-06-03

**Authors:** John Ontoy, Jobelle Bruno, Bishnu Shrestha, Jong Hyun Ham

**Affiliations:** 1 Department of Plant Pathology and Crop Physiology, Louisiana State University Agricultural Center, Baton Rouge, LA, United States; 2 Department of Plant Pathology and Crop Physiology, College of Agriculture, Louisiana State University, Baton Rouge, LA, United States

**Keywords:** bacterial panicle blight, genotyping-by-sequencing (GBS) of rice, host resistance, QTL mapping of rice, rice disease resistance, RNA-seq analysis of rice defense

## Abstract

**Introduction:**

Bacterial panicle blight (BPB) is a chronic disease of rice caused by the bacterial pathogen *Burkholderia glumae*. While rice varieties grown in the United States are mostly susceptible to BPB, a medium-grain variety Jupiter shows a moderate resistance phenotype. However, its underlying defense mechanisms and genetic traits are not known well.

**Methods:**

This study combined transcriptomic and genetic analyses to characterize the moderate BPB resistance of Jupiter. Comparative RNA-seq analysis of Jupiter and Bengal (a BPB susceptible medium-grain variety) was performed using panicle samples collected 24 hours post pathogen-inoculation at the flowering stage. We also conducted quantitative trait locus (QTL) mapping of the BPB resistance, using a recombinant inbred line (RIL) population from Jupiter and Bengal.

**Results and discussion:**

The results indicated that enhanced expression of genes involved in defense and stress response was associated with the partial resistance to BPB. Among the six QTLs identified for BPB resistance, *qBPB3.2* on Chromosome 3 was consistently detected across multi-year trials. Notably, this QTL was also located close to a major locus previously reported in studies using different mapping populations. The genes within these QTL regions included putative stress-related genes potentially linked to a network of pathogenesis-related and abiotic stress responsive regulators that were differentially expressed between the parents. Nevertheless, none of the candidate genes present in these genomic regions were identified as DEGs from the transcriptome analysis, suggesting their involvement in BPB resistance through unknown mechanisms beyond direct transcription in response to pathogen infection. This study provides valuable insights into environmentally responsive regulators and their complex interactions underlying resistance to BPB, highlighting the broad physiological impact of the pathogen infection on the host.

## 1 Introduction

Bacterial panicle blight (BPB) is an emerging rice disease in the United States and other rice growing countries around the world (Ham et al., 2011; Nandakumar et al., 2007). Since first reported in 1956 (Goto and Ohata, 1956), BPB has been reported in more than 18 countries across Africa, Asia, Latin America, and North America (Ortega and Rojas, 2021). The disease is mainly caused by the bacterium *Burkholderia glumae*, but other *Burkholderia* species, including *B. gladioli* and *B. plantarii*, also cause seedling blight and panicle blight in rice (Ham et al., 2011; Sayler et al., 2006; Nandakumar et al., 2009). Production of toxoflavin is an essential trait of the pathogen to induce the symptoms on rice seedlings and grains, and *B. glumae* can cause disease at any stage of the rice life cycle (Suzuki et al., 2004). In early stages of rice development, *B. glumae* can cause rotting in seeds before germination or stunting and chlorosis in seedlings, and during vegetative stages, the pathogen can cause lesions in leaves or sheath (Iiyama et al., 1995; Nandakumar et al., 2009). However, most devastating damages of this disease occur when the bacterium infects the panicles and interferes with grain development (Nandakumar et al., 2009; Wamishe et al., 2015). In severe cases, most of the panicles are left unfilled to remain erected, in contrast to healthy bending panicles due to filled grains (Nandakumar et al., 2009). BPB outbreaks occur mostly at the rice heading stage in conducive environmental conditions including prolonged high night temperatures (Ham et al., 2011).

BPB is considered as a seedborne disease, so infected seeds are the primary source of inoculum (Nandakumar et al., 2009; Zhu et al., 2008). Severe outbreaks of this disease occurred in the southeast United States during the mid- and late-1990’s and lately 2010 and 2011, resulting in up to 50% yield loss in the fields growing susceptible varieties across the southeastern United States (Nandakumar et al., 2009; Shahjahan et al., 2000; Mulaw, 2018) and approximately 10% to 20% yield losses in the Texas Rice Belt (Zhou et al., 2011). Global climate change could worsen BPB outbreaks because *B. glumae* can grow even at 41 °C and prolonged high temperatures are a conducive condition of this disease (Shew et al., 2019) (Saddler, 1994).

Management option for BPB is very limited due to several reasons. Few chemical control options are available, except that oxolinic acid has been used for BPB in some countries. However, this chemical is not allowed to use for managing BPB in the United States due to the frequent occurrence of resistant populations of the pathogen (Hikichi et al., 1989; Zhou, 2019). Although some *indica*-type rice genotypes were reported to be resistant to BPB (Mizobuchi et al., 2018), most *japonica* rice varieties are susceptible to the disease with only a few cultivars showing moderate resistance to BPB (Mizobuchi et al., 2016; Pinson et al., 2010; Zhou, 2019). In the United States, a medium-grain (temperate *japonica*) variety Jupiter has shown a moderate resistance phenotype in BPB, according to numerous field trials with artificial pathogen inoculation (Zhou, 2019; Ontoy et al., 2023; Sha et al., 2006).

Genetic studies on BPB have indicated that rice disease resistance to the disease is conferred by quantitative trait loci (QTLs). However, quantitative disease resistance to BPB is highly dependent on environmental conditions, and the high variability in disease phenotypes depending on experimental systems has hindered genetic study to understand resistance to BPB and breeding efforts to improve disease-resistant traits (Mizobuchi et al., 2016). In our previous study, we identified *qBPB3.1* as the most significant QTL associated with the BPB phenotype (Ontoy et al., 2023). However, its close linkage to the days-to-heading trait complicates interpretation as the effect of the QTL on disease phenotype may be confounded by pleotropic effects related to heading time.

In this study, we performed RNA-seq analysis and genetic mapping to identify candidate genes and QTLs associated with BPB phenotypes, using two temperate *japonica* (medium-grain) varieties, Jupiter and Bengal, and their RIL population. These rice varieties represent contrasting BPB resistance but share similar days-to-heading traits, allowing us to identify BPB-related genes and QTLs without confounding pleiotropic effects from different heading times of the two rice genotypes. To further characterize the genetic basis of BPB resistance in Jupiter, we also integrated RNA-seq analysis with QTL-linkage mapping of the parental lines, leading to identification of key defense mechanisms and underlying genetic elements contributing to BPB resistance.

## 2 Materials and methods

### 2.1 Plant materials, growth conditions, and inoculation

Seeds of the medium-grain rice varieties Jupiter (moderate resistant to BPB) (Sha et al., 2006) and Bengal (susceptible to BPB) (Linscombe et al., 1993) were surfaced sterilized, germinated, and grown in a greenhouse (28-32 °C and 16 h light/8 h dark) until panicle formation. The resistant and susceptible phenotypes of these varieties were previously confirmed in trials at the H. Rouse Caffey Rice Research Station (Rayne, LA, USA). Three fully exposed panicles from each cultivar were inoculated with the virulent *B. glumae* strain 336gr-1. For inoculation, *B. glumae* 336gr-1 was cultured on Luria Broth (LB) agar media for 2 days at 28 °C, scraped, and resuspended in 10mM MgCl_2_ to a final concentration of OD_600_ = 0.1 (~ 1x10^8^ CFU/ml). Panicles were spray-inoculated using a hand sprayer, and plants were maintained under greenhouse conditions (28-32 °C, 80-90% relative humidity). Panicle samples from both Jupiter and Bengal were collected at 0, 6 and 24 hours post-inoculation (hpi), along with mock inoculated controls (10 mM MgCl_2_). All samples were flash-frozen in liquid nitrogen and stored at -80 °C until RNA extraction.

### 2.2 RNA isolation and high-throughput DNA sequencing

Total RNA was extracted using the RNeasy Plant Mini Kit (Qiagen, Germantown, MD, USA) following the manufacturer’s protocol. RNA quality and quantity were assessed using a nanodrop spectrophotometer (ND-1000 Thermo Fisher Scientific, Wilmington, DE, USA). RNA samples from Jupiter and Bengal collected at 0 hpi, 6 hpi, and 24 hpi were submitted to the Pennington Biomedical Research Center Genomics Core Facility for RNA-seq analysis. Single-end reads (75 bp per read) were generated on an Illumina Hi-Seq platform. A customized pipeline was employed for quality assessment, adapter trimming, removal of low-quality reads, alignment to the reference genome, and differential expression analysis between Jupiter and Bengal across time points. The pipeline included Trimmomatic v0.32 (Bolger et al., 2014), STAR v2.7.10a (Dobin et al., 2013), featureCounts v1.6.1 (Liao et al., 2014), and DESeq2 v1.36.0 (Love et al., 2014). Trimmomatic was used to remove the adapter sequences and trim reads with quality scores (Q) below 30.

High-quality processed reads were aligned to the International Rice Genome Sequence Project (IRGSP) pseudomolecules (version 7) of the reference genome Nipponbare using STAR with default parameters. Gene-level counts were generated with featureCounts, aligning only to exons. The filtered count table was used for downstream analyses. DESeq2 was used to normalize the sample counts through the regularized log (rlog) transformation. Pairwise analysis between genotypes within each inoculation state and across time points was also performed using DESeq2. This module aggregated read counts from all replicates under different conditions and computed fold change values between two conditions. Differentially expressed loci with an adjusted p-value of ≤ 0.05 were considered significant.

For downstream analyses, IDEP version 1.1 (Ge et al., 2018), TBtools version 1.120 (Chen et al., 2020), Cytoscape version 3.9.1 (Shannon et al., 2003), and R-based packages were employed to generate heatmap, figures for differentially expressed genes (DEGs), gene networks, and ID conversions. Data set comparisons to identify novel, unique and shared loci were performed using DiVenn version 1.2 (Sun et al., 2019) and TBtools version 1.120 (Chen et al., 2020), producing Venn diagrams and upset plots. Chromosomal mapping of DEG locations was generated with ShinyGO version 0.77 (Ge et al., 2020). Weighted Gene Co-expression Network Analysis (WGCNA) (Langfelder and Horvath, 2008) was applied using RNA-seq count matrices to detect gene clusters (modules) and construct gene networks, enabling identification of modules highly correlated with external sample traits.

### 2.3 Gene enrichment and pathway analysis

Gene enrichment and pathway analyses were conducted to categorize DEGs based on biological processes, cellular components, and molecular functions across different timepoints for both Jupiter and Bengal using ShinyGO (v0.77) (Ge et al., 2020) and GenekitR (v1.0.5) (Liu and Li, 2023). To identify key pathways under each condition, pathway analysis of significant DEGs was performed using Mapman (v3.5.1) (Thimm et al., 2004).

### 2.4 Reverse transcription quantitative PCR

Eleven differentially expressed genes (DEGs) were selected based on their functional classification and differential expression patterns (**Table 1**). Candidate genes were prioritized from MapMan (v3.5.1) (Thimm et al., 2004) binning categories associated with receptors and biotic stress–related pathways. Specifically, genes were selected based on their contrasting expression profiles between the cultivars Jupiter and Bengal, where expression in Jupiter was significantly higher or absent in Bengal at 6 hpi, which was identified as the critical timepoint for differential defense activation. Five to six seedlings were grown individually in pots under greenhouse conditions at the LSU AgCenter Doyle Chambers Central Research Station (Baton Rouge, LA). Three fully exposed panicles from each cultivar were inoculated with the pathogen as previously described. Each sampling point included three biological replications. Samples were collected at 0, 3, 6 and 24 hpi and immediately frozen in liquid nitrogen. Timepoint 3 hpi was included to investigate the possibility that these candidate DEGs exhibit early expression responses prior to 6 hours post-inoculation, thereby allowing a more accurate characterization of their initial expression patterns. Total RNA was extracted from tissues using the Direct-zol RNA kit (Zymo Research, Tustin CA), and first-strand cDNA was synthesized with the iScript™ cDNA Synthesis Kit (Bio-Rad Laboratories, Hercules, CA, USA) following the manufacturer’s instructions. Primers (**Supplementary Table 1**) were designed using the IDT PrimerQuest tool (https://www.idtdna.com/pages/tools/primerquest). RT-qPCR was performed using SYBR Green (Bio-Rad) on a Bio-Rad CFX96 real-time PCR system following the manufacturer’s protocol. Amplification conditions were: 95 °C for 30s, followed by 40 cycles of 95 °C for 5s and 57 °C for 30s. Expression levels were normalized to the rice reference gene *Actin*. Three biological replicates were analyzed per gene, and relative expression was calculated using the Livak method (2^-ΔΔCt^) (Livak and Schmittgen, 2001).

**Table 1:** List of differentially expressed loci/genes between Jupiter and Bengal selected for validation through RT-qPCR based on their predicted functions in the biotic response pathway.

UGA Rice Locus	Class	Gene name (gene symbol) in RAP-DB
LOC_Os07g44450	stress.biotic.PR-proteins	Similar to dirigent-like protein
LOC_Os04g44470	stress.biotic.PR-proteins. proteinase inhibitors. trypsin inhibitor	Alpha-amylase/subtilisin inhibitor (*RASI, OsEnS-69*); Defense of the seed against microorganisms
LOC_Os09g25060	RNA. regulation of transcription	WRKY GENE 76 (*OsWRKY76, OsWRKY76.1, OsWRKY76.2, OsWRKY76.3*); WRKY transcription factor, Transcriptional repressor, Pathogen defense, Alkali tolerance at the germination stage
LOC_Os06g45140	RNA. regulation of transcription	RICE SEED B-ZIPPER 5 (*OsbZIP52, bZIP52, OsbZIP52/RISBZ5, RISBZ5*); Basic leucine zipper (bZIP) transcription factor, Negative regulator of cold and drought stress response
LOC_Os05g01140	hormone metabolism. salicylic acid. synthesis-degradation	JASMONIC ACID CARBOXYL METHYLTRANSFERASE 1 (*OsJMT1*); jasmonic acid carboxyl methyltransferase, Regulation of plant development and herbivory-induced defense response, Resistance to brown planthopper (BPH)
LOC_Os02g48770	hormone metabolism. salicylic acid. synthesis-degradation	JASMONIC ACID CARBOXY METHYL TRANSFERASE 2 (*JMT-2, OsJMT-2, OsJMT2*); SAM dependent carboxyl methyltransferase family protein
LOC_Os03g14180	stress. abiotic. heat	26 KDA HEAT SHOCK PROTEIN (*OsHSP26, Oshsp26, OsHSP26.7, HSP26.7*); Similar to Heat shock protein 26
LOC_Os04g01740	stress. abiotic. heat	Heat shock protein 90.1 (*Hsp90, OsHSP90.1*); Similar to Heat shock protein 82. (Os04t0107900-01); Heat shock protein 81-1 (*HSP81-1*) (Heat shock protein 83). (Os04t0107900-02); Similar to Heat shock protein 80. (Os04t0107900-03); Similar to Heat shock protein 82
LOC_Os03g16020	stress. abiotic. heat	17.3 KDA CLASS I HEAT SHOCK PROTEIN (*OsHsp17.3, OsHSP17.3, Oshsp17.3*); 17.4 kDa class I small heat shock protein, Heat tolerance (Os03t0266900-02)
LOC_Os01g08860	stress. abiotic. heat	18.0 KDA CLASS II HEAT SHOCK PROTEIN (*OsHSP18.0-CII, Oshsp18.0-CII, OsHSP18.0, OsHSP18.2*); Class II small heat shock protein, Positive regulation in both *Xoo* resistance and heat/salt tolerance
LOC_Os01g53220	stress. abiotic. heat	HEAT STRESS TRANSCRIPTION FACTOR C1b, Heat shock factor C1b, Heat stress transcription factor C1b, Heat stress transcription factor C-1b, Heat stress transcription factor 3 (*HSFC1B, OsHsfC1b, HsfC1b, HSF03, OsHsf-03, OsHSF3, HSF3, HSF11, rHsf11*)

### 2.5 Development of Bengal x Jupiter RIL mapping population

A recombinant inbred line (RIL) population was developed through single-seed descent from a cross between Bengal, a susceptible medium-grain variety (Linscombe et al., 1993), and Jupiter, a moderately resistant medium-grain variety to bacterial panicle blight (Sha et al., 2006). Seeds from individual panicle of each F_2_ plant were advanced as separate lines in successive generations. The final F_7_ RIL population, consisting of 300 lines, was evaluated for BPB resistance and days-to-50% heading (DTH) under field conditions at the LSU AgCenter H. Rouse Caffey Rice Station (Rayne, LA and greenhouse conditions at the LSU AgCenter Doyle Chambers Central Research Station (Baton Rouge, LA) during 2019, 2020, 2021 and 2023 growing seasons.

### 2.6 Phenotypic evaluation of RIL population for BPB and days-to-heading (DTH)

*B. glumae* 336gr-1, a virulent strain of the pathogen (Karki et al., 2012), was used as the inoculum. Each rice plant was sprayed in the field with a bacterial suspension (~1 x 10^8^ CFU/ml; OD_600_ = 0.1) prepared in 10 mM MgCl_2_ using a hand sprayer. Inoculation began when 30 - 50% of plants in test plots were headed. Due to asynchronous flowering, inoculations were repeated at least three times at 2 – 4-day intervals. For the 2019 and 2020 growing seasons, two replications (one row having 15–20 plants per replication) were used, while three replications were implemented in 2021 and 2023. BPB resistance was evaluated 14 days post-inoculation by scoring panicle discoloration on a 0–9 scale: 0 = no symptoms and 9 = > 90% symptomatic panicle area (Shahjahan et al., 2000)(**Supplementary Figure 1**). Lower scores (0–4) represent resistant to moderately resistant phenotypes with minimal panicle discoloration, intermediate scores (5–6) indicate moderate susceptibility, and higher scores (7–9) represent susceptible phenotypes with severe panicle discoloration and a high proportion of symptomatic spikelets. Days-to-50% heading (DTH) were recorded in 2019 and 2023 as the date when 50% of plants in a plot were headed.

### 2.7 Genotyping-by-sequencing and data processing

From the 300 RILs evaluated for BPB phenotype, 186 lines along with the parental cultivars (cvs. Jupiter and Bengal) were selected for genotyping-by-sequencing (GBS). Leaf tissue was collected from each RIL and parent, and DNA was extracted using the Qiagen DNeasy Plant Mini Kit (Qiagen Inc., Valencia, CA, USA) following the manufacturer’s protocol. DNA quality was assessed by electrophoresis on a 2% agarose gel and quantified using a nanodrop spectrophotometer (ND-1000 Thermo Fisher Scientific, Wilmington, DE, USA). Approximately 50 ng of DNA per line was submitted to University of Minnesota Genomics Center (https://genomics.umn.edu/) for purification, library preparations, and high-throughput DNA sequencing on a NextSeq 1 x 150 high output flow cell, generating ~ 1.9 million reads per sample. Sequence quality was evaluated using FastQC version 0.11.5 (Andrews, 2010).

Data processing combined GATK and TASSEL pipelines. Processed reads were aligned to the International Rice Genome Sequence Project (IRGSP) pseudomolecules version 7 of the Nipponbare japonica reference genome using BWA-MEM with default parameters (Li, 2013). SAM files were sorted and converted to BAM using Picard tools, and variants were called using GATK Haplotypecaller (Poplin et al., 2017). The resulting variant call file (VCF) was filtered in TASSEL (Bradbury et al., 2007) to remove low quality calls (Q < 25), set minor allele frequency (MAF) to 0.05, retain biallelic SNPs, and convert to HapMap format, then transformed into a genotypic matrix suitable for QTL analysis using IciMapping software v. 4.2.53 (www.isbreeding.net/software). RILs identified as self-fertilized progeny of either parent were removed, resulting in a final set of 164 RILs used for linkage map construction and QTL analysis.

### 2.8 Statistical analysis

Analysis of variance (ANOVA) and broad sense heritability (h^2^) were calculated using the AOV functions in QTL IciMapping v4.2.53 (www.isbreeding.net/software). Histogram was generated using packHV package in R (https://rdrr.io/cran/packHV/man/hist_boxplot.html). Correlations between phenotypic traits were computed using Pearson’s correlation coefficients for each year in R (Team, 2018). Broad-sense heritability for each trait and year was estimated using the formula described by Holland (2002) (Holland et al., 2003).

### 2.9 QTL mapping

Genotypic data derived from GBS were used to construct the linkage map showing the physical positions of SNPs. Adjacent markers with identical alleles were assumed to represent uniform chromosome segments, disregarding potential double recombination within the interval. Linkage maps were generated using QTL IciMapping v4.2.53 (www.isbreeding.net/software) and combined with phenotypic data to identify QTLs associated with target traits. QTL mapping was performed using the inclusive composite interval mapping for additive QTL (ICIM-ADD) method, with a logarithm of odds (LOD) threshold of 2.0 determined by 1, 000 permutations. SNP density maps illustrating QTL positions were created using SRplot (https://www.bioinformatics.com.cn/srplot). The position and effect of each QTL were estimated, and genes within selected QTL intervals were retrieved from the Rice Genome Annotation Project Database (Release 7; https://rice.uga.edu/). Non-parametric QTL analysis was conducted using R/qtl v.1.48–1 package (Broman et al., 2003) with 1, 000 permutations to assess significance. Gene enrichment and localization analyses were performed on genes within selected genomic regions using ShinyGO v0.77 (Ge et al., 2020) at a false discovery rate (FDR) of 0.05, identifying significant GO terms and candidate genes potentially associated BPB resistance and days to 50% heading.

## 3 Results

### 3.1 Transcriptome profiling of Jupiter and Bengal at the early stage of BPB infection

**Figure 1:**
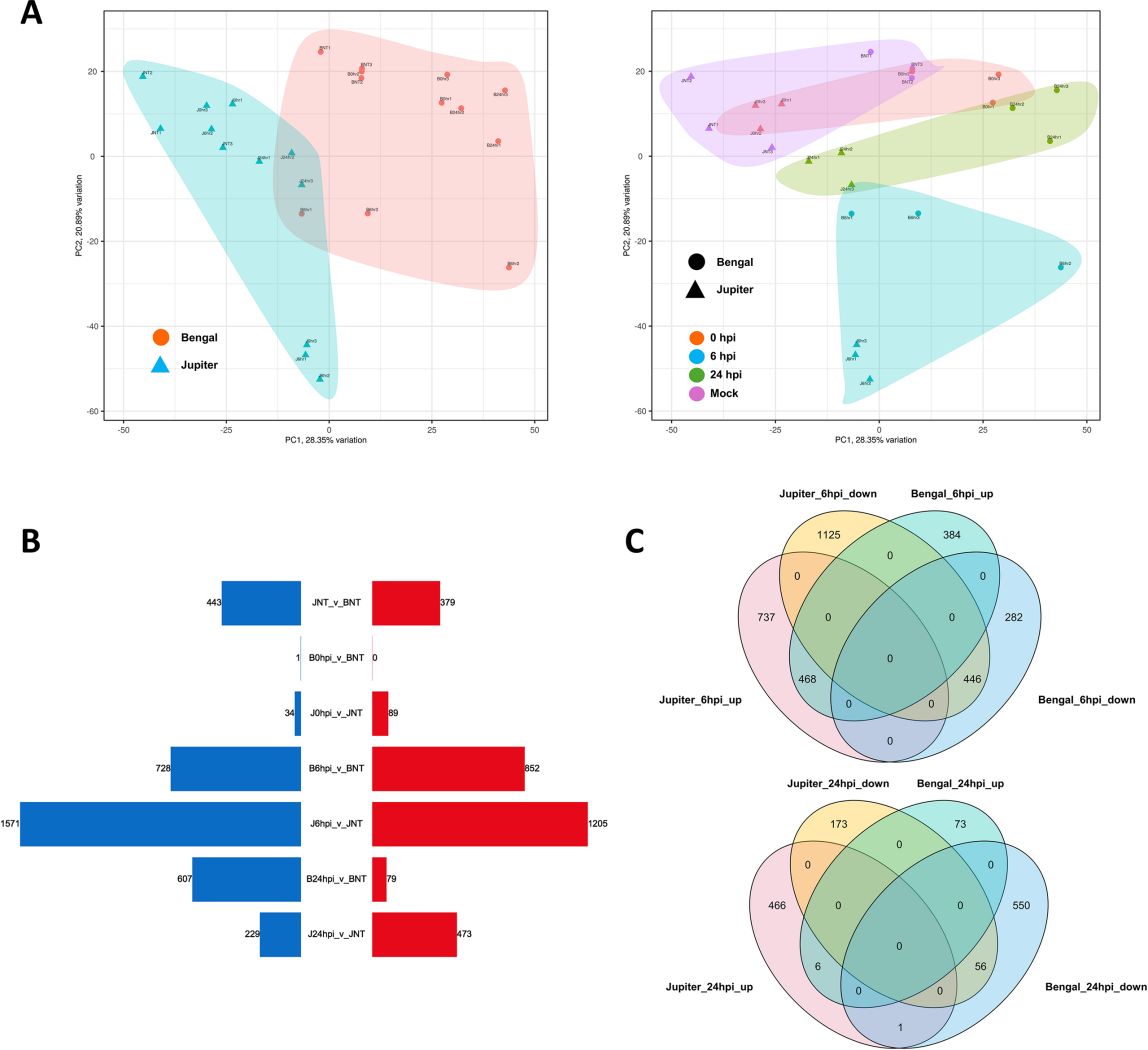
Transcriptional response of rice cultivars Jupiter and Bengal during early infection by *Burkholderia glumae*. **(A)** principal component analysis (PCA) of rlog-transformed RNA-seq read counts showing transcriptome separation among samples. PC1 (explaining the largest proportion of variance) significantly separates samples by cultivar (Jupiter vs. Bengal; p = 5.09 × 10^-5^), indicating strong genotype-dependent transcriptional differences. PC2 significantly separates samples by infection timepoint (0, 6, and 24 hpi; p = 4.63 × 10^-6^), reflecting dynamic temporal responses to *B. glumae*. **(B)** Differentially expressed genes (DEGs) over time. Bar plots summarize the total number of DEGs identified in each cultivar at 0, 6, and 24 hpi relative to their respective controls. Red bars indicate DEGs upregulated, whereas blue bars indicate DEGs downregulated. **(C)** Shared and cultivar-specific DEGs depicting overlapping and unique DEGs between Jupiter and Bengal at 6 and 24 hpi.

Sequencing of Bengal (susceptible) and Jupiter (moderately resistant) across 0-, 6-, and 24-hours post-inoculation (hpi) produced 6.22 – 21.06 million raw reads per sample, of which 85.7 – 92% aligned to the *Oryza sativa* Nipponbare IRGSP-1.0 reference genome, and 5.13 - 16.76 million reads mapped to exonic regions after filtering (**Supplementary Figure 2**). Differential expressions were assessed within each cultivar by comparing inoculated samples to their respective uninoculated controls, generating genome-wide expression profiles across all rice chromosomes. Principal component analysis (PCA) revealed strong genotype- and time-dependent transcriptional signatures: PC1 (28.35%) separated samples by cultivar, whereas PC2 (20.89%) separated samples by time post-inoculation (**Figure 1A**). At the default statistical threshold (adjusted p ≤ 0.05), Bengal exhibited 1, 1580, and 686 DEGs at 0, 6, and 24 hpi, respectively, whereas Jupiter showed 123, 2776, and 702 DEGs at corresponding timepoints (**Figure 1B**).

### 3.2 Differential expression between Jupiter and Bengal

Both cultivars exhibited minimal transcriptional changes at 0 hpi, indicating limited basal differences prior to infection. The strongest transcriptional reprogramming occurred at 6 hpi, consistent with the timepoint containing the highest number of DEGs in both genotypes (**Figure 1B**). At this timepoint, 446 downregulated and 468 upregulated genes were shared between Bengal and Jupiter, these numbers declined by 24 hpi to 56 downregulated and 6 upregulated shared DEGs (**Figure 1C**). Bengal showed no upregulated genes at 0 hpi, increasing to 852 at 6 hpi but decreasing to 79 by 24 hpi, while downregulated genes increased from 1 at 0 hpi to 728 and 607 at 6 and 24 hpi, respectively (**Figure 1B**). In Jupiter, 89 genes were upregulated at 0 hpi, rising to 1, 205 at 6 hpi and decreasing to 473 by 24 hpi; downregulated genes numbered 34, 1, 571, and 229 at the respective timepoints (**Figure 1B**). Across equivalent timepoints, Jupiter consistently showed a larger number of DEGs than Bengal.

**Figure 2:**
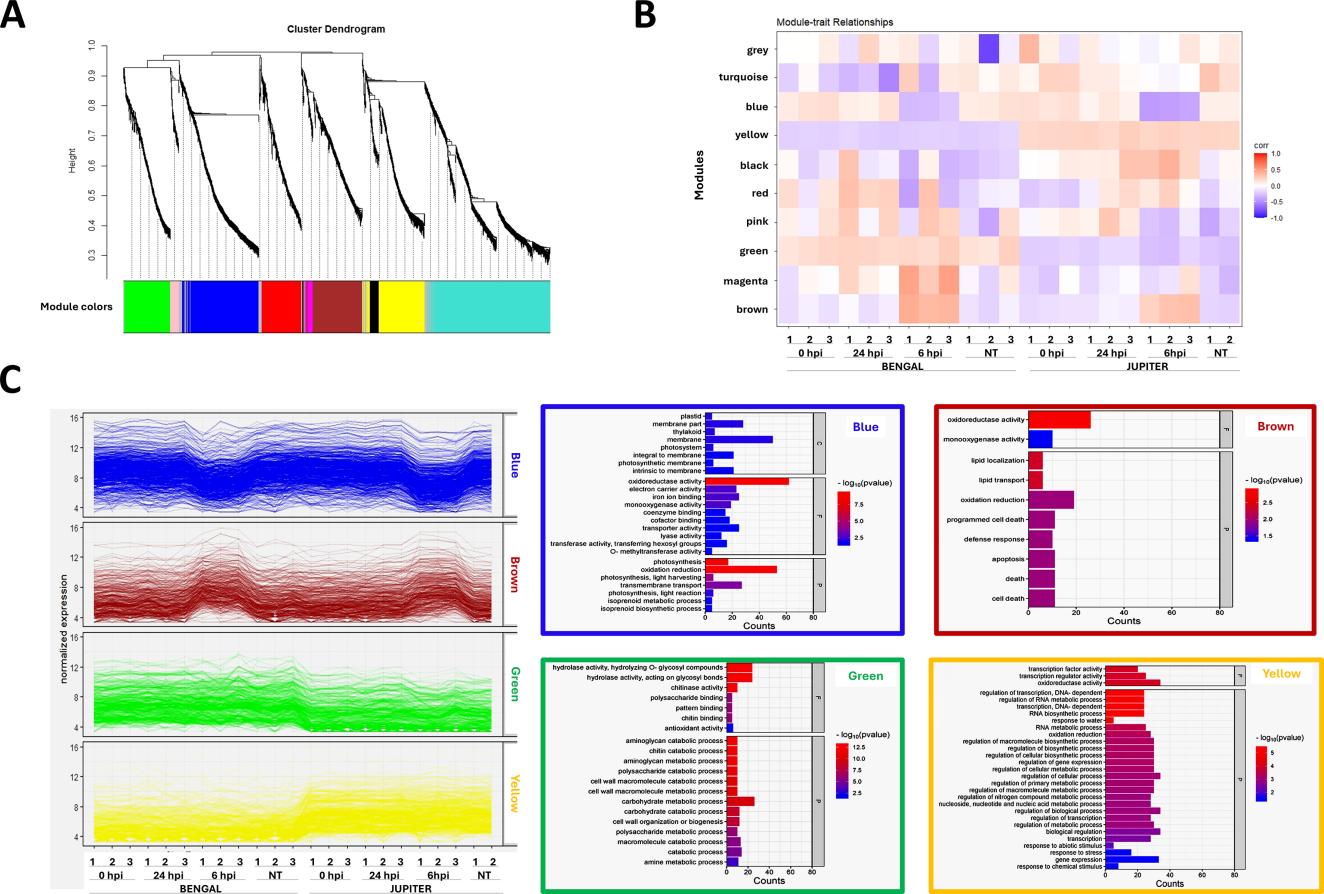
Gene network modules associated with cultivar and infection stage (hours after *B. glumae* inoculation) identified by weighted gene co-expression network analysis (WGCNA). **(A)** The cluster dendrogram exhibiting modules represented by unique color labels, which reflect groups of genes with similar expression patterns and potential shared biological functions. **(B)** The heatmap that shows correlations between module eigengenes (representing the overall expression profile of each module) and experimental traits, including cultivars (Bengal and Jupiter), infection stages (0, 6, and 24 hpi), and non-inoculated controls (NT). Red indicates positive correlation, while blue indicates negative correlation between module expression and the respective trait. The intensity of the color reflects the strength of the correlation. This analysis highlights modules specifically associated with early (6 hpi) and later (24 hpi) infection stages, as well as cultivar-dependent transcriptional responses. **(C)** Gene ontology (GO) enrichment analysis performed for genes within each co-expression module to infer their putative biological roles. Enriched functional categories reveal distinct biological processes represented by individual modules, including defense response, signaling pathways, metabolic reprogramming, and stress-related processes.

Weighted gene co-expression network analysis (WGCNA) revealed cultivar-specific modules, including a Jupiter-associated yellow module and a Bengal-associated green module, while blue and brown modules represented timepoint-specific clusters enriched at 6 hpi in both varieties (**Figures 2A–C**). Functional predictions showed that the blue module contained genes involved in isoprenoid metabolism, plastid and thylakoid function, photosynthesis, and transport (**Figure 2C**). The Jupiter-enriched yellow module was dominated by genes related to transcriptional regulation, redox processes, stress responses, and metabolic regulation (**Figure 2C**). The brown module, highly expressed at 6 hpi in both cultivars, contained genes associated with defense activation, oxidoreduction, and programmed cell death (**Figure 2C**). The Bengal-associated green module was enriched for carbohydrate metabolism and catabolic pathways (**Figure 2C**).

### 3.3 Functional analysis of DEGs in response to BPB

**Figure 3:**
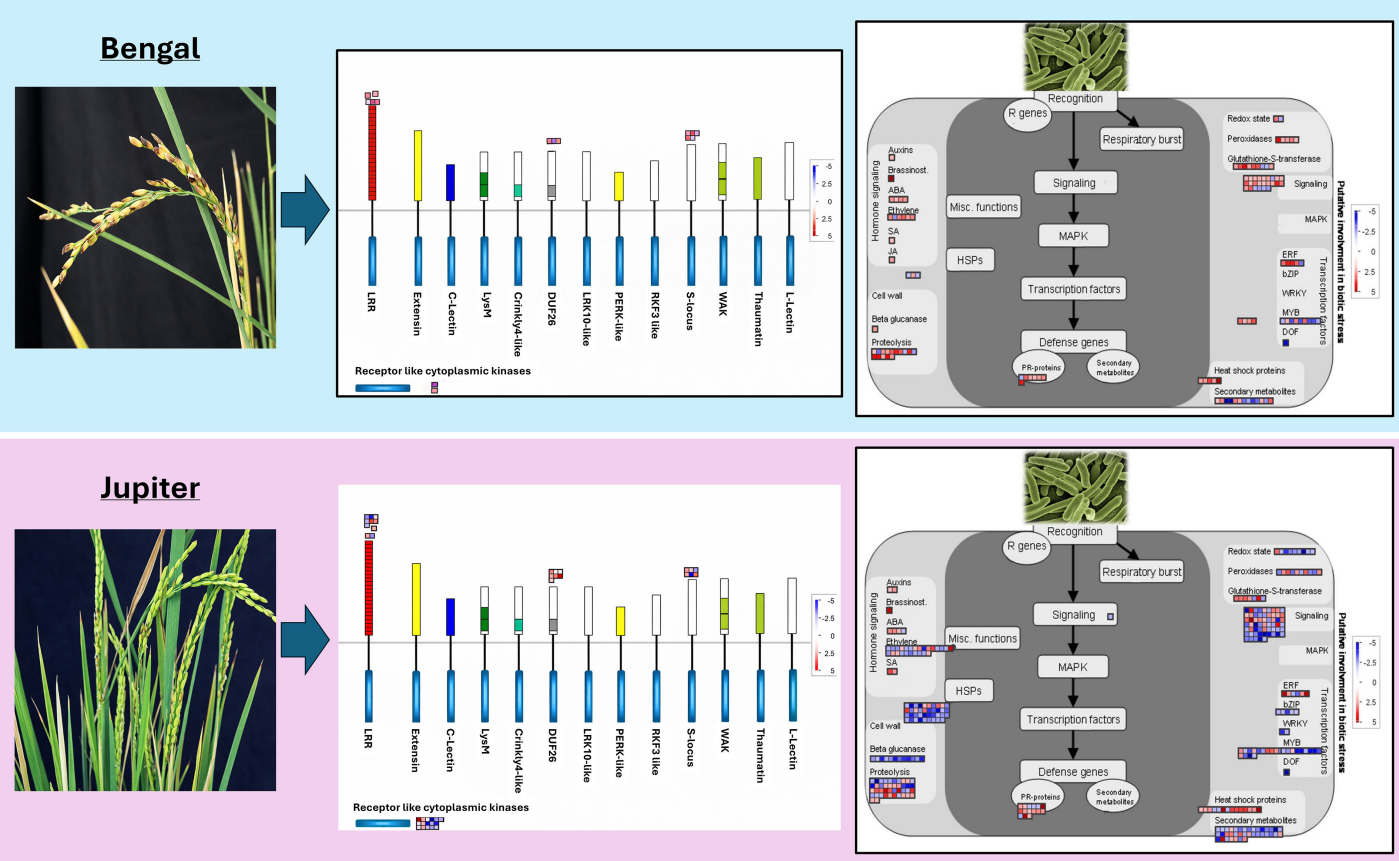
Functional categorization of differentially expressed genes (DEGs) involved in pathogen recognition and biotic stress responses in Bengal and Jupiter during early infection by *Burkholderia glumae*. Significantly differentially expressed genes (adjusted p-value ≤ 0.05, FDR; |log_2_ fold change| ≥ 2.0) at 6 hours post-inoculation (hpi) were classified into functional categories associated with pathogen perception and defense signaling. Genes were grouped based on annotated biological function, and their log_2_ fold change values are displayed. Red indicates upregulated genes, and blue indicates downregulated genes. The boxes next to the panicle images present differential expression patterns of genes for receptor and receptor-like kinases involved in pathogen recognition and early immune signaling. The panels on the right-side present defense signaling and stress-response pathways and DEGs involved in these functions.

Functional and pathway analyses were focused on 6 hpi, as this timepoint exhibited stronger transcriptional reprogramming across multiple analyses compared to 24 hpi (**Figures 1**, **2**). Both varieties activated genes associated with pathogen recognition—including NB-LRR genes and receptor-like cytoplasmic kinases—and core stress response pathways. Jupiter consistently exhibited a larger number of DEGs across major functional categories than Bengal, particularly genes associated with abiotic stress, heat shock factors/proteins (HSFs/HSPs), homeostasis, and hormone signaling (**Figure 3**). Enriched functional classes at 6 hpi included signaling cascades, transcription factor activity, reactive oxygen species (ROS) regulation, production of pathogenesis-related (PR) proteins, and biosynthesis of secondary metabolites (**Figure 3**).

Both cultivars activated oxidative stress–relief components such as glutathione-S-transferases and peroxidases, though expression of these loci diminished by 24 hpi (**Supplementary Figures 3**, **4**). Jupiter displayed a higher number of oxidative stress–related DEGs, although the number of strongly upregulated ROS genes (≥ 2 log_2_FC) was similar between cultivars at 6 hpi (six genes each) (**Supplementary Figure 4**). Glutathione-S-transferase genes were largely absent by 24 hpi in both cultivars, while peroxidase-related loci decreased in Jupiter but increased in Bengal at 24 hpi, suggesting genotype-specific ROS modulation (**Supplementary Figure 4**). Despite Bengal displaying strong respiratory burst signatures, these responses did not translate into effective resistance.

Jupiter showed stronger early activation of cell wall–related genes at 6 hpi, including genes involved in precursor synthesis, structural proteins, wall-modifying enzymes, and esterases (**Supplementary Figure 4**). Five cell wall–associated genes were upregulated in Jupiter compared with two in Bengal at 6 hpi; this pattern reversed by 24 hpi, when Bengal exhibited more upregulation. Notably, LOC_Os02g40260, encoding a leucine-rich repeat/extensin family protein, was uniquely upregulated in Jupiter at 6 hpi (**Supplementary Figure 4**). Jupiter also displayed more DEGs linked to MYB transcription factors regulating secondary cell wall biogenesis.

Signaling pathways–including MAPK cascades, LRR receptors, calcium sensors, G-proteins, and phosphoinositide-related genes–were strongly activated at 6 hpi in both genotypes but more prominently in Jupiter. By 24 hpi, signaling activity sharply diminished; only one stress-related DIR domain protein remained significantly upregulated in Jupiter, whereas most signaling genes in Bengal were downregulated (**Supplementary Figure 5**). PR proteins were strongly induced at 6 hpi and returned to near-basal levels by 24 hpi in both cultivars (**Figure 3**; **Supplementary Figures 3**, **5**). Jupiter and Bengal displayed comparable numbers of PR genes at 6 hpi, but Jupiter exhibited much higher expression of several key loci (**Supplementary Figure 5**). One of the strongest responses in Jupiter was LOC_Os07g44450 (5.17 log_2_FC), which showed no significant induction in Bengal. Bengal’s most upregulated PR gene (LOC_Os11g30210; 3.9 log_2_FC) was expressed at an even higher level in Jupiter (4.9 log_2_FC). Several NB-LRR genes exhibited genotype-specific induction patterns. Notably, LOC_Os04g58080 showed higher expression at 6 hpi in Jupiter compared to Bengal, along with other genes such as LOC_Os01g52880, LOC_Os04g44470 and LOC_Os03g63150, which were also more highly expressed in Jupiter at both 6 and 24 hpi. In contrast, a distinct set of NB-LRR genes, including LOC_Os06g47800, LOC_Os03g40194 and LOC_Os12g11930, displayed elevated expression in Bengal (**Supplementary Figure 5**). Transcription factor activity peaked at 6 hpi, with Jupiter exhibiting a larger set of TF-related DEGs than Bengal. WRKY, MYB, ERF, and bZIP families showed strong induction, although most declined by 24 hpi (**Figure 3**; **Supplementary Figures 3**, **5**). ERF genes were strongly upregulated in both cultivars, while WRKY and MYB genes showed stronger induction in Jupiter (**Supplementary Figure 5**). Notably, Bengal increased WRKY and bZIP expression sharply at 24 hpi rather than at the early timepoint (**Supplementary Figure 4**). Two bZIP TFs (LOC_Os06g45140 and LOC_Os01g11350) were markedly downregulated in Jupiter from 6 to 24 hpi (**Supplementary Figure 5**).

Hormone-related defense signaling also peaked at 6 hpi (**Figure 3**). Jupiter showed stronger induction of genes associated with salicylic acid, ethylene, and brassinosteroid pathways, whereas Bengal displayed relatively stronger activation of jasmonic acid–related genes. Both cultivars significantly upregulated the brassinosteroid-related gene LOC_Os05g12040, with Bengal showing slightly higher expression (**Supplementary Figure 6**). Secondary metabolite biosynthesis genes, including flavonoid, isoprenoid, and phenylpropanoid pathways—were strongly activated at 6 hpi in both cultivars (**Figure 2**; **Supplementary Figure 6**). Bengal showed more upregulated genes, while Jupiter showed more downregulated genes in this category (**Supplementary Figure 6**). Jupiter strongly upregulated LOC_Os01g51980 (tryptophan aminotransferase), whereas Bengal highly expressed LOC_Os08g07100 (terpene synthase) (**Supplementary Figure 6**). A lignin-biosynthesis gene (LOC_Os09g23540) was uniquely upregulated in Jupiter (**Supplementary Figure 6**). HSFs/HSPs were markedly enriched in Jupiter at 6 hpi, where nine HSF-related DEGs exhibited 2–6.9 log_2_FC induction, compared with four in Bengal (**Figure 3**; **Supplementary Figure 6**). Jupiter also maintained HSF activity at 24 hpi, whereas Bengal showed complete suppression (**Supplementary Figures 3**, **6**). Several HSF genes (e.g., LOC_Os03g14180, LOC_Os04g01740, LOC_Os03g16020) remain significantly upregulated in Jupiter at 24 hpi (**Supplementary Figures 3**, **6**). Protein degradation–related genes showed similar numbers of upregulated loci in both cultivars, but Jupiter exhibited a larger set of significantly perturbed genes overall at both 6 and 24 hpi (**Supplementary Figure 6**).

Gene Ontology (GO) enrichment of differentially expressed genes (DEGs) revealed distinct temporal and cultivar-specific responses to *B. glumae* infection. In the biological process (BP) category, both varieties showed early activation (6 hpi) of stress-related pathways; however, Jupiter exhibited stronger enrichment for response to temperature stimulus, oxidative stress, and hormone-mediated signaling, while Bengal showed greater induction of responses to toxins, xenobiotic stimuli, and defense-associated metabolic processes (**Supplementary Figure 7**). By 24 hpi, Jupiter shifted toward processes related to protein folding, cellular homeostasis, and regulation of biosynthetic pathways, whereas Bengal maintained elevated enrichment for secondary metabolite biosynthesis and immune-related metabolic responses (**Supplementary Figure 8**). For molecular function (MF), Jupiter displayed enhanced enrichment of oxidoreductase activity, peroxidase activity, and carbohydrate-active enzymes, consistent with a rapid detoxification response (**Supplementary Figure 7**). Bengal, in contrast, showed late enrichment of transmembrane transporter activity, ATP-binding functions, and catalytic activities associated with redox metabolism, indicating a delayed metabolically driven defense strategy over time (**Supplementary Figures 7**, **8**). In the cellular component (CC) category, DEGs in Jupiter were enriched for membrane-bounded organelles, chloroplast components, and mitochondrial structures, particularly at 24 hpi, suggesting alterations in energy metabolism and intracellular signaling (**Supplementary Figures 7**, **8**).

### 3.4 Validation using reverse transcription quantitative PCR

**Figure 4:**
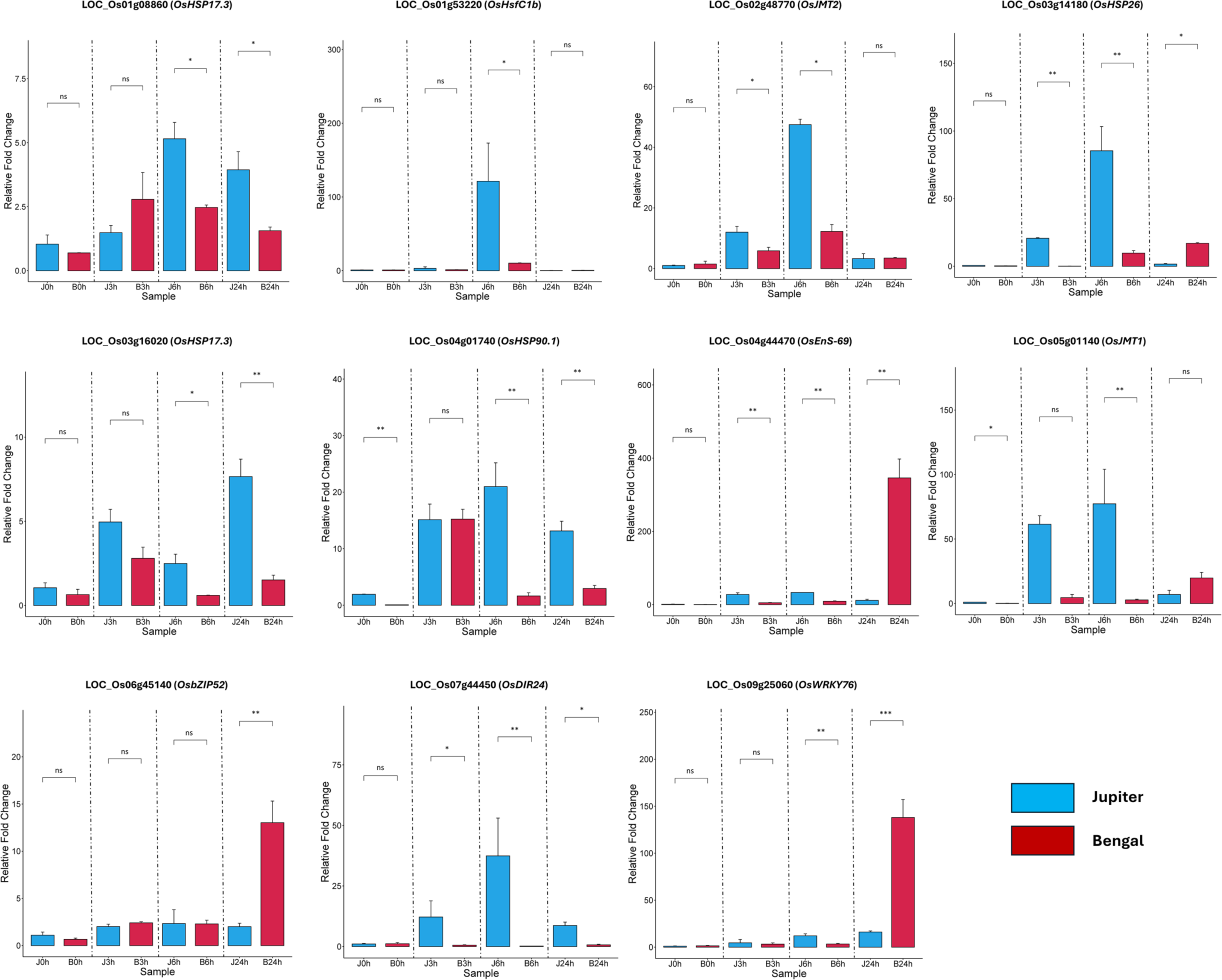
The reverse transcription quantitative PCR (RT-qPCR) analysis with selected defense- and stress-related genes for validation of the transcriptome analysis. Relative gene expression levels were quantified by RT-qPCR for selected candidate genes representing key functional categories, including pathogenesis-related (PR) proteins, abiotic stress response, transcriptional regulation, and hormone signaling/metabolism. Expression levels were normalized to the internal reference gene and calculated using the 2^-ΔΔCt^ method. Fold changes are shown relative to the respective non-treated control samples. The analyzed genes include heat shock proteins (LOC_Os01g08860, LOC_Os03g14180, LOC_Os03g16020 and LOC_Os04g01740), a heat shock transcription factor (LOC_Os01g53220), defense-related genes (LOC_Os07g44450 and LOC_Os04g44470), a jasmonic acid (JA) signaling-related gene (LOC_Os05g01140 and LOC_Os02g48770), and a transcription factors including bZIP (LOC_Os06g45140) and WRKY (LOC_Os09g25060). * = statistically significant at p < 0.05 (less than 5% probability the result occurred by chance). ** = statistically significant at p < 0.01 (less than 1% probability the result occurred by chance). *** = highly statistically significant at p < 0.001 (less than 0.1% probability the result occurred by chance). ns = not significant (no statistically meaningful difference detected).

To validate RNA-seq expression profiles, 11 DEGs representing defense functions were analyzed by RT-qPCR (**Table 1**). An additional early timepoint (3 hpi) was included to capture rapid responses. Two PR-protein related genes LOC_Os07g44450 (*OsDIR24*) and LOC_Os04g44470 (*OsEnS-69*) were induced early at 3 and 6 hpi in the moderately resistant cultivar Jupiter, while LOC_Os04g44470 was substantially upregulated at 24 hpi in the susceptible cultivar Bengal (**Figure 4**). Regulatory genes encoding transcriptional factors, LOC_Os09g25060 (*OsWRKY76*) and LOC_Os06g45140 (*OsbZIP52*), also showed early induction in Jupiter at 6 hpi and delayed strong expression in Bengal at 24 hpi like the expression pattern of LOC_Os04g44470 (**Figure 4**). Defense/stress-responsive genes involved in the jasmonic acid (JA) metabolism, LOC_Os05g01140 (*OsJMT1*) and LOC_Os02g48770 (*OsJMT2*), were more strongly expressed in Jupiter at 3 and 6 hpi before declining at 24 hpi, whereas their expression was lower and delayed in Bengal (**Figure 4**). All validated genes for heat shock proteins, LOC_Os01g53220 (*OsHsfC1b*), LOC_Os01g08860 (*OsHSP17.3*), LOC_Os03g14180 (*OsHSP26*), LOC_Os03g16020 (*OsHSP17.3*), and LOC_Os04g01740 (*OsHSP90.1*), were strongly induced in Jupiter at 6 hpi, with some of them maintaining high expression levels through 24 hpi (**Figure 4**). Taken together, RT-qPCR data were consistent with RNA-seq results, revealing early and sustained expression of key defense genes in the resistant cultivar Jupiter, while the susceptible cultivar Bengal exhibited delayed and less coordinated defense activation.

### 3.5 BPB phenotype of the RIL population

**Figure 5:**
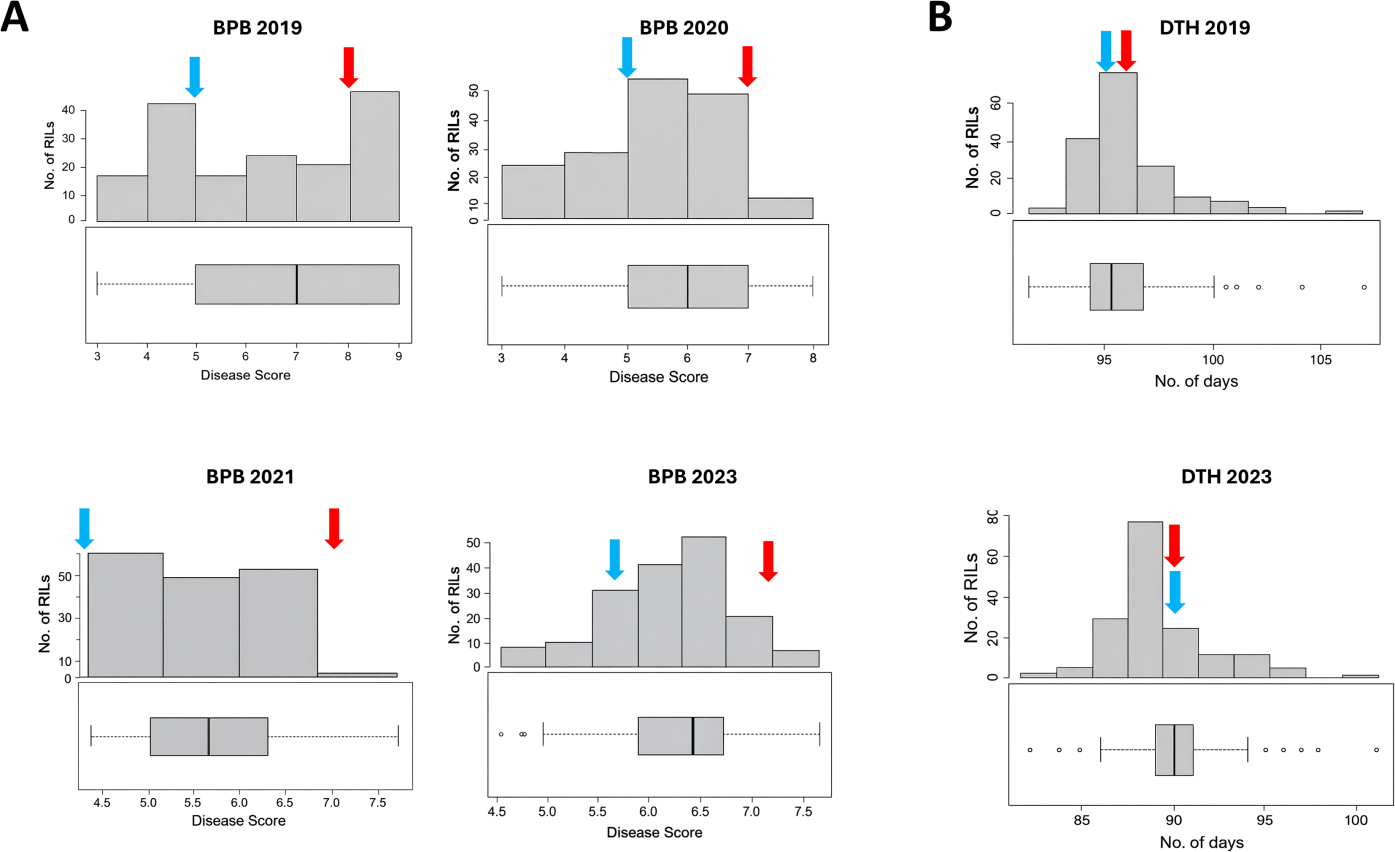
Distribution of phenotypes in bacterial panicle blight (BPB) disease severity and days-to-50% heading among recombinant inbred lines (RILs) derived from the cross Bengal × Jupiter across multiple field environments. **(A)** BPB disease scores evaluated in field trials conducted in 2019, 2020, 2021, and 2023. **(B)** Days-to-50% heading (DTH) measured in 2019 and 2023. The x-axis represents disease severity scores for BPB evaluations and number of days to 50% heading, while the y-axis indicates the number of RILs observed in each class. Blue and red arrows indicate the phenotypes of parent cultivars Jupiter and Bengal, respectively.

Jupiter and Bengal significantly differed in reaction to BPB, and the RIL population derived from them also showed phenotypic variability in BPB, as well as in days-to-50% heading (DTH) (**Figure 5**; **Table 2**). Jupiter consistently exhibited resistant to moderately resistant responses to BPB across multiple years of field evaluations, whereas Bengal consistently displayed susceptible phenotypes (**Figure 5**; **Table 2**). For Jupiter, the mean BPB values for 2019, 2020, 2021 and 2023 in the field were 5.0, 5.0, 3.67 and 5.7, respectively. For Bengal, they were 8.0, 7.0, 7.0 and 7.2, respectively (**Table 2**). The mean BPB values of the 164 RILs for corresponding years/tests were 6.7, 5.9, 5.7 and 6.4, respectively (**Table 2**). In case of the DTH trait for 2019 and 2023, the mean of the RILs, 95.7 and 90.2 days, was close to the DTH values of both parents, where Jupiter was 90–95 days and Bengal was 90-96.5 days (**Table 2**). Traits were not normally distributed in RILs (**Figure 5**; **Supplementary Table 2**). The distribution also showed the presence of transgressive segregants (**Figure 5**). There was a variation in heritability values (h^2^) computed on a family mean basis (**Table 2**). The heritability values ranged from 0.43 to 0.77 for the BPB disease index and 0.70 to 0.79 for the DTH (**Table 2**), suggesting moderate heritability in both traits.

**Table 2:** Mean values and ranges of phenotypic traits of the parents (cvs. Bengal and Jupiter) and recombinant inbred lines (RILs) for bacterial panicle blight (BPB) and days-to-50% heading (DTH).

Trait name	Mean Jupiter/Bengal	RILs mean	RILs range	Std. Dev.^b^	RILs, F-values^c^	Heritability^d^
BPB2019_FIELD	5.0/8.0 *	6.73	3.0-9.0	1.89	4.41***	0.7733
BPB2020_FIELD	5.0/7.0*	5.91	3.0-8.0	1.21	2.32***	0.5690
BPB2021_FIELD	3.7/7.0**	5.69	3.67-7.67	0.79	1.75***	0.4275
BPB2023_FIELD	5.7/7.2*	6.40	4.56-7.67	0.65	2.09***	0.5212
DTH2019	95.0/96.50 ^ns^	95.69	91-107	2.49	3.35***	0.6980
DTH2023	90.33/90.33 ^ns^	90.22	82-101	2.77	4.82***	0.7926

^a^ T-test between the means of Jupiter and Bengal.

^b^ Standard deviations of RILs.

^c^ ANOVA of RILs.

^d^ Broad sense heritability computed on family mean basis (per year).

^ns^ Not significant.

* Significant at P < 0.05.

** Significant at P < 0.01.

*** Significant at P < 0.001.

### 3.6 Correlation analysis of traits

Correlation analysis revealed weak to moderate associations among the traits evaluated; however, several statistically significant relationships were observed across years for both BPB disease scores and days-to-50% heading (DTH) (**Table 3**). BPB disease scores recorded in different field environments showed weak correlations across years, ranging from 0.19 to 0.3. For example, BPB scores in 2020 were positively correlated with those in 2019 (r = 0.23), 2021 (r = 0.19), and 2023 (r = 0.30).

**Table 3:** The Pearson’s correlation matrix of bacterial panicle blight (BPB) and days to 50% heading (DTH) traits in RILs.

	BPB2019 FIELD	BPB2020 FIELD	BPB2021_FIELD	BPB2023 FIELD	DTH 2019	DTH 2023
BPB2019 FIELD	1.000					
BPB2020 FIELD	0.2302**	1.000				
BPB2021 FIELD	0.1974**	0.1924**	1.000			
BPB2023 FIELD	0.0200^ns^	0.3020***	0.2060**	1.000		
DTH 2019	-0.6148***	-0.1913**	-0.1466*	0.0280ns	1.000	
DTH 2023	-0.3690***	-0.0347^ns^	0.0142^ns^	0.2584^ns^	0.5716***	1.000

*Significant at P < 0.05.

**Significant at P < 0.01.

***Significant at P < 0.001.

^ns^ Not significant.

Between BPB and DTH traits, BPB severity generally showed a negative relationship with DTH, suggesting that earlier heading lines tend to be more susceptible to BPB. However, these correlations were weak and often not statistically significant (**Table 3**). Although the strongest negative association between BPB scores and DTH was observed in the field trial of 2019 (r = −0.62), a significant positive correlation between BPB and DTH was detected in only 50% of the cases (4 out of 8 combinations), indicating that BPB is largely independent of from DTH (**Table 3**).

### 3.7 QTL analysis of BPB and DTH

**Figure 6:**
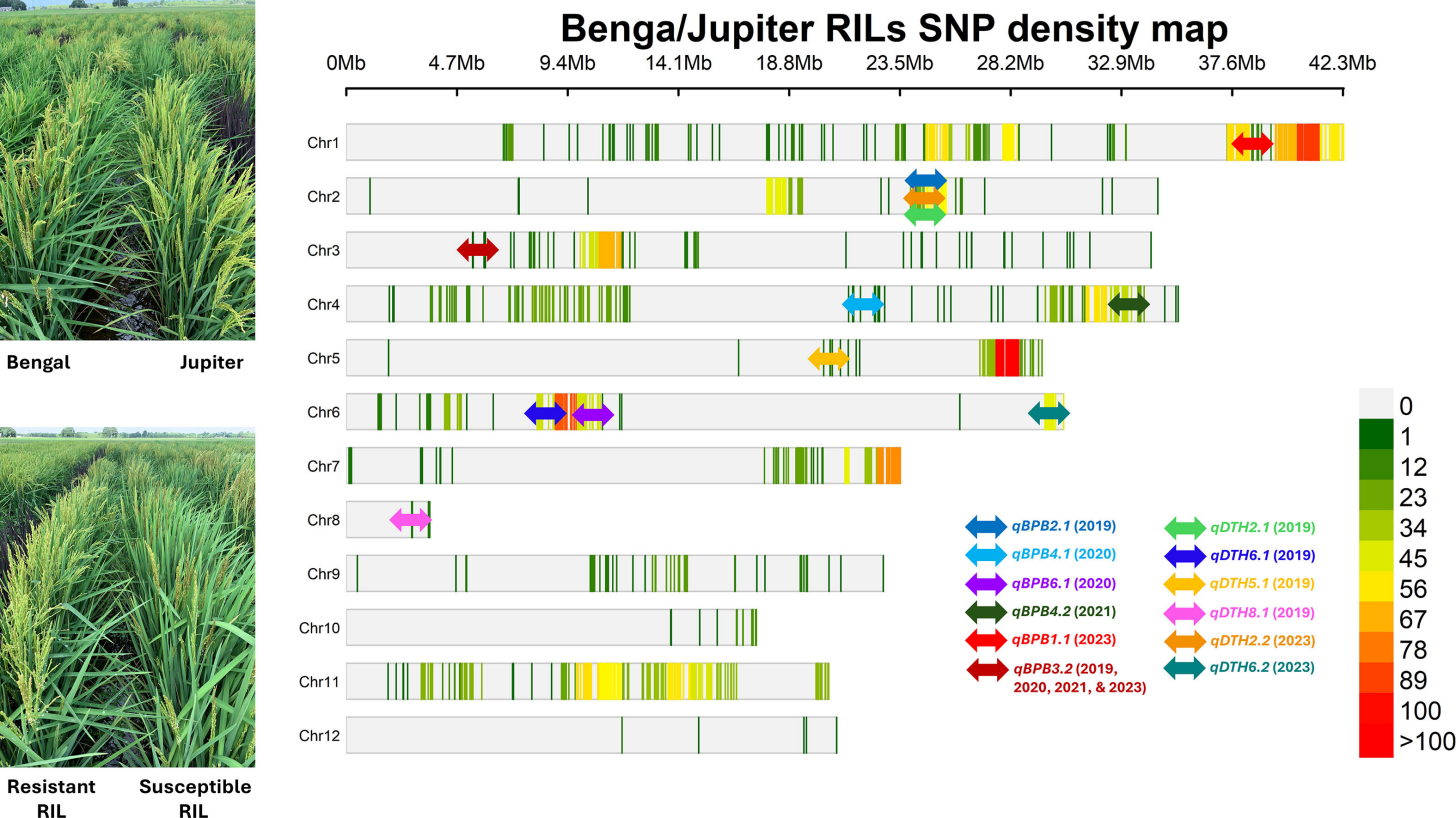
A SNP density and genetic linkage map of the Bengal × Jupiter recombinant inbred line (RIL) population showing quantitative trait loci (QTLs) associated with bacterial panicle blight (BPB) resistance and days-to-50% heading (DTH). The horizontal bars represent individual chromosomes scaled according to their physical length (Mb), with genomic positions indicated along the top axis. Vertical colored lines within each chromosome represent SNP markers, and their color intensity corresponds to SNP density per genomic window as indicated by the color scale (right panel), ranging from low density (dark green) to high density (red; >100 SNPs per window). Regions lacking color represent genomic intervals with few or no SNP markers. QTLs detected for bacterial panicle blight resistance (*qBPB*) and days to 50% heading (*qDTH*) are indicated by colored arrows positioned along the corresponding chromosomal locations. Each arrow denotes the approximate physical interval of a QTL detected across different years, with colors distinguishing individual loci and the associated trait category. The QTLs presented in this image are *qBPB2.1* (2019), *qBPB4.1* (2020), *qBPB6.1* (2020), *qBPB4.2* (2021), *qBPB1.1* (2023), and *qBPB3.2* (detected across 2019, 2020, 2021, and 2023) for bacterial panicle blight resistance, and *qDTH2.1* (2019), *qDTH5.1* (2019), *qDTH6.1* (2019), *qDTH8.1* (2019), *qDTH2.2* (2023), and *qDTH6.2* (2023) for heading date. QTL detection was performed using inclusive composite interval mapping (ICIM) implemented in QTL IciMapping software v.4.2.53.

QTL analyses were conducted using phenotypic data of field trials for the Bengal × Jupiter RIL population, including four years of BPB evaluations (2019, 2020, 2021, and 2023) and two years of day-to-50% heading (DTH) data (2019 and 2023). QTL detection was performed using the inclusive composite interval mapping (ICIM) method. A total of twelve QTLs, including one common BPB QTL (*qBPB3.2*) and two closely adjacent DTH QTLs (*qDTH2.1* and *qDTH2.2*), were identified with the phenotype data from all field trials (**Table 4**; **Figure 6**). From the field evaluation of BPB in 2019, two QTLs were identified on Chromosome (Chr) 2 and 3. The QTL found in Chr 2 was a major one with 10.74% phenotypic variance explained (PVE) in the population, while the one found in Chr 3 only causing 8.05% variation in the population (**Table 4**; **Figure 6**). In these QTLs, the favorable allele for the major one was Bengal, while that for the minor one was Jupiter. From the BPB data in 2020, three QTLs were identified on Chr 3 (*qBPB3.2*), Chr 4 (*qBPB4.1*) and Chr 6 (*qBPB6.1*) (**Table 4**; **Figure 6**). These QTLs were minor QTLs with PVE ranging from 7.86 to 9.38% (**Table 4**). The favorable allele for *qBPB3.2* was Jupiter, while Bengal allele was desirable for the *qBPB4.1* and *qBPB6.1*. BPB data of 2021 revealed two minor QTLs located on Chr 3 and Chr 4, respectively (**Table 4**; **Figure 6**). These QTLs contributed PVE ranging from 5.46 to 5.52% within the RIL population, where the QTL on Chr 3 was contributed by Jupiter and the other one on Chr 4 was from Bengal (**Table 4**). From the BPB data of 2023, two QTLs were detected from Chr 1 and Chr 3, which had PVE ranging from 8.31 to 9.06% (**Table 4**; **Figure 6**). All the favorable allele for these QTLs were contributed by Jupiter. Among the 4 years of BPB evaluation in the field, *qBPB3.2* was consistently detected in all four years, though its effect was minor, having PVE ranging from 5.46 (in 2021) to 8.31% (in 2023) (**Table 4**).

**Table 4:** The QTLs detected for the traits related to bacterial panicle blight (BPB) and days to 50% heading (DTH) in the RILs by inclusive composite interval mapping.

Trait	QTL	Ch	Position (cM)	Marker interval	LOD	PVE (%)	Additive effect	
BPB2019 FIELD	*qBPB2.1*	2	183	SNP24516990-SNP24517028	3.6191	10.7433		-0.5380
	*qBPB3.2*	3	1	SNP5390402-SNP5859102	2.7997	8.0524		0.4742
BPB2020 FIELD	*qBPB3.2*	3	1	SNP5390402-SNP5859102	2.8345	7.9112		0.3138
	*qBPB4.1*	4	122	SNP22849919-SNP24012086	3.2603	9.2094		-0.3310
	*qBPB6.1*	6	203	SNP11694250-SNP26043651	3.3724	9.4597		-0.3364
BPB2021 FIELD	*qBPB3.2*	3	1	SNP5390402-SNP5859102	2.1007	5.4565		0.1933
	*qBPB4.2*	4	330	SNP33326877-SNP33600878	2.1248	5.5213		-0.1896
BPB2023 FIELD	*qBPB1.1*	1	434	SNP38723937-SNP38847770	4.2975	9.0695		0.2024
	*qBPB3.2*	3	1	SNP5390402-SNP5859102	4.1615	8.3124		0.1912
DTH2019	*qDTH2.1*	2	178	SNP24278397-SNP24278688	7.7382	15.6102		0.9907
	*qDTH5.1*	5	109	SNP20978712-SNP21310082	3.8100	7.2722		0.6774
	*qDTH6.1*	6	78	SNP9205762-SNP9209514	3.4078	6.5199		-0.6383
	*qDTH8.1*	8	0	SNP2813748-SNP3501665	3.7788	7.1468		-0.6696
DTH2023	*qDTH2.2*	2	182	SNP24387239- SNP24387241	4.9961	12.0867		0.9380
	*qDTH6.2*	6	272	SNP30104177- SNP30450927	2.8695	6.9223		-0.7141

For DTH, six QTLs were identified to be associated with the trait, which were on Chr 2, Chr 5, Chr 6, and Chr 8 (**Figure 6**). Two closely adjacent QTLs on Chr 2 (*qDTH2.1* and *qDTH2.2*) were major QTLs having PVE of 15.61% and 12.09%, respectively (**Table 4**). The rest four QTLs showed relatively less PVE values for the DTH trait; *qDTH5.1*, *qDTH6.1*, *qDTH6.2*, and *qDTH8.1* were 7.27%, 6.52%, 6.90%, and 7.15%, respectively (**Table 4**). No QTLs were found to be commonly associated with both BPB and DTH traits. Although *qBPB2.1*, *qDTH2.1*, and *qDTH2.2* are positioned in close proximity on Chr 2, they do not occupy the same genomic region (**Table 4**; **Figure 6**).

### 3.8 Non-parametric analysis due to nonnormality of distribution of the traits

The traits for BPB and DTH did not follow normality of the phenotypic distribution for QTL analysis (**Figure 5**; **Supplementary Table 2**). To improve efficiency, non-parametric analysis (Kruskal-Wallis’ test) of markers was conducted to show association to the traits of interest.

A total of nine significant positions with their associated markers were identified (**Table 5**). From the BPB field data of 2019, three positions were identified located in Chr 2, Chr 3 and Chr 5 with their corresponding associated markers SNP24692491, SNP5369525, and SNP27616764, respectively, with LOD scores ranging from 2.91 to 4.28 (**Table 5**). The position found in Chr 3 overlapped to *qBPB3.2* from the previous analysis, while the position on Chr 2 was closely adjacent to the *qBPB2.1* (**Table 4**). From the BPB field data of 2020, only one position was detected in Chr 3 with the associated marker SNP5369525, but its LOD score of 2.80 was not significant (**Table 5**). However, this site overlapped to the *qBPB3.2* that was previously identified from the QTL-linkage analysis (**Table 4**). This non-parametric analysis also identified positions overlapping with *qBPB3.2* from the BPB field data of 2021 and 2023 with corresponding LOD of 2.62 (not statistically significant) and 4.13 and with a closely associated marker SNP5859102 (**Table 5**). The locus on Chr 3 that overlaps with *qBPB3.2* was consistently detected in the BPB field evaluations conducted in 2019, 2020, 2021, and 2023.

**Table 5:** Markers associated with bacterial panicle blight (BPB) and days to 50% heading (DTH) identified by non-parametric analysis.

Trait	Position^a^	Marker	Kruskal-Wallis’ test P value	LOD score
BPB2019_FIELD	Ch2 (loc 187.2)	SNP24692491	0.001***	4.28
	Ch3 (loc 0)	SNP5369525	0.046*	2.91
	Ch5 (loc 151.5)	SNP27616764	0.009**	3.49
BPB2020 FIELD	Ch3 (loc 0)	SNP5369525	0.061^ns^	2.80
BPB2021 FIELD	Ch3 (loc 1.68)	SNP5859102	0.129 ^ns^	2.62
BPB2023 FIELD	Ch3 (loc 1.68)	SNP5859102	0.002**	4.13
	Ch4 (loc 336.58)	SNP33612619	0.015*	3.31
DTH2019	Ch2 (loc 178)	SNP24278688	0.000***	5.38
	Ch5 (loc 147)	SNP26898079	0.000***	4.96
DTH2023	Ch1 (loc 152)	SNP20178856	0.003**	4.19
	Ch2 (loc 182)	SNP24504717	0.000***	5.82

^a^ Ch (chromosome), loc # (genetic location associated with a trait).

* Significant at P < 0.05.

** Significant at P < 0.01.

*** Significant at P < 0.001.

^ns^ not significant.

For the DTH trait, four positions were identified in Chr 1, Chr 2 and Chr 5 with associated markers SNP20178856, SNP24278688, SNP24504717 and SNP26898079, having LOD scores of 4.19, 5.38, 5.82 and 6.29, respectively (**Table 5**). The two DTH-related positions identified from Chr 2 overlapped and closely adjacent to *qDTH2.1* that was previously identified while the position identified from Chr 5 was adjacent to the *qDTH5.1* that was also previously identified through inclusive composite interval mapping (**Table 4**).

Markers associated with these significant positions were evaluated based on their effect on the genotypes and showed significant changes relative to alleles of Bengal (AA) and Jupiter (BB) (**Supplementary Figure 9**). In the SNP24692491 marker in Chr 2, which was closely adjacent to *qBPB2.1*, the presence of Bengal allele decreased the disease index while Jupiter allele increased it (**Supplementary Figure 9**). The associated markers for the position in Chr 3 that overlapped to *qBPB3.2* were SNP5369525 and SNP5859102. The presence of Bengal allele was associated with the higher disease index (**Supplementary Figure 9**). For the associated marker in Chr 6 detected from the BPB field evaluation in 2020, the Bengal allele at SNP26043651 was associated with reduced disease score, while the Jupiter allele with increased disease score (**Supplementary Figure 9**). For the DTH, the Bengal alleles in the associated markers SNP24278688 in Chr 2 and SNP26898079 in Chr 5 tend to be associated with longer DTH while the Jupiter allele with shorter DTH (**Supplementary Figure 9**).

### 3.9 Gene annotation and enrichment analysis of the QTLs for BPB and DTH

**Figure 7:**
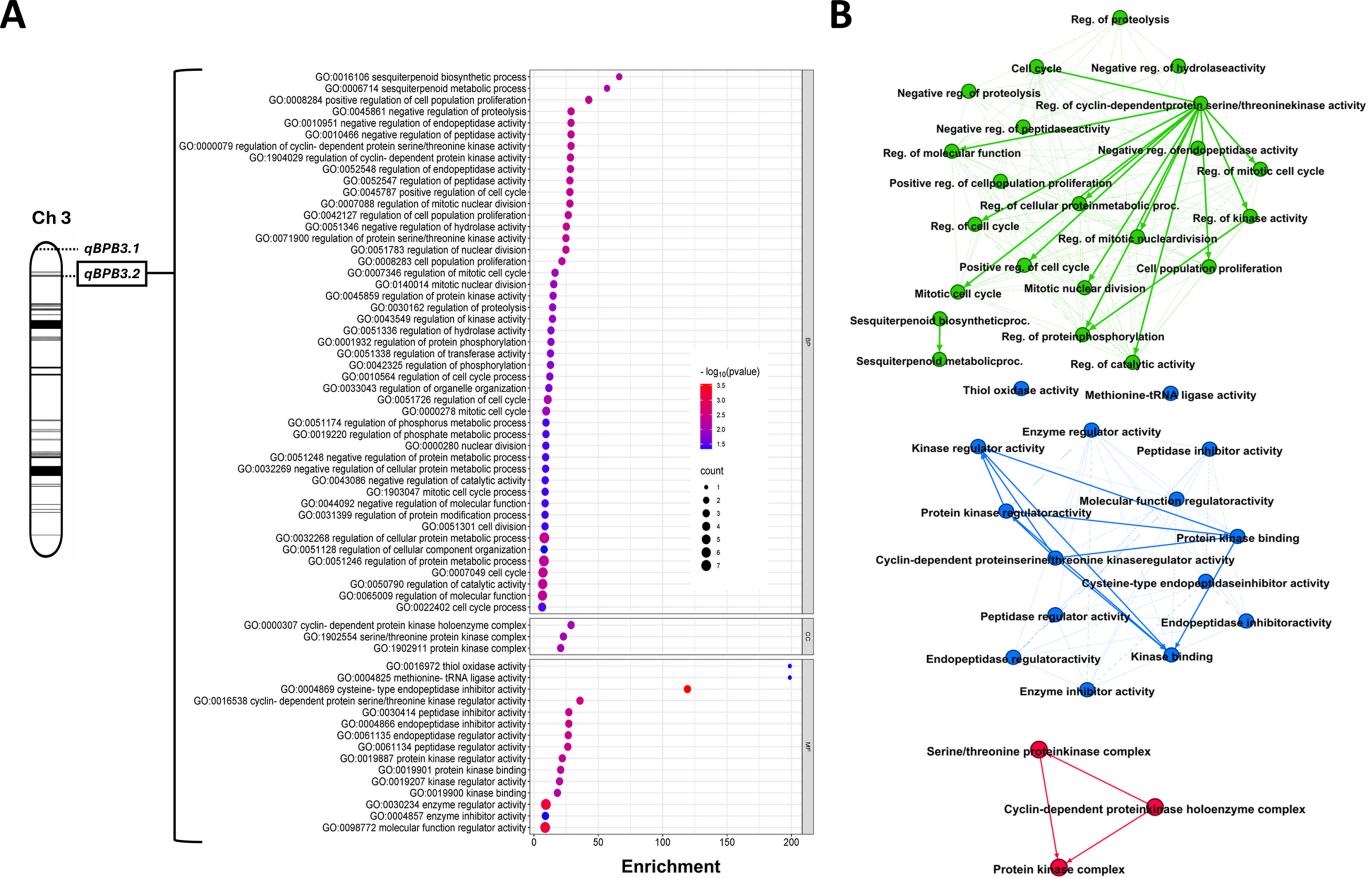
Functional and network analysis (FDR ≤ 0.05) of genes associated with *qBPB3.2* and its position relative to *qBPB3.1*. **(A)** The approximate physical positions of *qBPB3.2* (identified in this study with a RIL population from a Bengal/Jupiter cross) and *qBPB3.1* [identified in our previous study (Ontoy et al., 2023] with a RIL population from a Trenasse x Jupiter cross), along with the result of enrichment analysis of genes located within the *qBPB3.2* interval. Enriched GO terms are displayed according to enrichment score on the x-axis. Dot size represents the number of genes associated with each GO term, while dot color corresponds to statistical significance (−log10 p-value). The analysis identified significant enrichment for biological processes related to cell cycle regulation, mitotic nuclear division, regulation of protein phosphorylation, regulation of kinase activity, proteolysis regulation, and cellular metabolic processes, as well as molecular functions associated with kinase binding, enzyme regulator activity, and peptidase inhibitor activity. **(B)** Functional interaction networks of significantly enriched GO terms grouped by ontology category: biological processes (green), molecular functions (blue), and cellular components (red). Nodes represent enriched GO terms, while edges indicate functional relationships based on shared genes. The network highlights interconnected modules dominated by cell-cycle regulation, kinase-mediated signaling, and protease regulatory pathways, suggesting that genes within the *qBPB3.2* region may influence bacterial pod blight resistance through regulatory mechanisms involving protein phosphorylation, kinase activity, and proteolytic control.

Further downstream analysis was performed on the genomic regions for one common QTLs for BPB (*qBPB3.2*) and three QTLs for DTH (*qDTH2.1*, *qDTH2.2* and *qDTH5.1*), which were detected by both inclusive composite interval mapping and the non-parametric analysis. Referring to UGA Rice annotation file (http://rice.uga.edu/), *qBPB3.2*, flanked by SNP5390402 and SNP5859102, contained 78 genes. Enrichment analysis of these 78 genes predicted 18 biological process-related pathways, 10 molecular-related pathways, and 12 cellular component-related pathways associated with these loci at FDR ≤0.05 with their related genes (**Figure 7**; **Supplementary Tables 3**-**5**). Seven genes were predicted to be associated to defense and stress response: LOC_Os03g11160, LOC_Os03g11170, LOC_Os03g11180, LOC_Os03g10780, LOC_Os03g11220, LOC_Os03g11140 and LOC_Os03g10750 (**Table 6**).

**Table 6:** List of candidate genes identified from the QTLs for BPB resistance and DTH found in chromosomes 2, 3, and 5.

UGA Rice Locus	Gene name (gene symbol) in RAP-DB^1^
qBPB3.2	
LOC_Os03g11160	cysteine proteinase inhibitor precursor
LOC_Os03g11170	cysteine proteinase inhibitor precursor
LOC_Os03g11180	like cystatin 6
LOC_Os03g10780	flap endonuclease, putative
LOC_Os03g11220	keratin, type I cytoskeletal 9, putative
LOC_Os03g11140	RhoGAP domain containing protein
LOC_Os03g10750	SPOTTED LEAF 35 (*OsSPL35*); CUE (coupling of ubiquitin conjugation to ER degradation) domain-containing protein, regulation of cell death and defense response
qDTH2.1/qDTH2.2/qDTH5.1	
LOC_Os02g40240	LEAF PANICLE 2, Leaf Panicle 2, LEAF PANICLE2 (*OsLP2, LPS*)
LOC_Os02g40100	Protein of unknown function DUF869
LOC_Os05g41450	DR1 ASSOCIATED PROTEIN 2 (*OsDrAp2, DrAp2, OsHAPL1, HAPL1, DLN150, OsDLN150*); histone-like transcription factor, transcriptional repressor, repressor of heading date, inhibition of flowering under long-day condition
LOC_Os05g44180	FLOWERING TIME LIKE GENE 10 (*OsFTL10, FTL10*); homolog of Hd3a, promotion of flowering, drought tolerance
LOC_Os05g41070	b-ZIP TRANSCRIPTION FACTOR 42 (*BZIP42, OsbZIP42, OsABF7, ABF7, HBF1, DLN149, OsDLN149*); bZIP transcription factor, modulation of the floral transition, floral repressor

^1^ Rice Annotation Project Database (https://rapdb.dna.affrc.go.jp/).

For DTH, two closely adjacent QTLs, *qDTH2.1* and *qDTH2.2*, were identified in Chr 2 and one from Chr 5 (**Figure 6**; **Table 4**). The *qDTH2.1* and *qDTH2.2* had a combined region of 0.12 Mb and containing 33 annotated genes and enrichment predicted 22 biological process-related pathways and 7 molecular-related pathways at FDR ≤0.05 (**Supplementary Tables 6**, **7**). Several candidate genes predicted to function in signaling pathway, such as LOC_Os02g40240 (*OsLP2*) and LOC_Os02g40100 (a DUF869 domain protein), were identified in this region as potential contributors to the trait (**Table 6**). In *qDTH5.1*, because the ICIMapping and non-parametric analysis detected a very closed regions, the original region was expanded from 20, 978, 712 bp to 26, 898, 079. It contained 991 annotated genes classified into significant GO terms subdivided into 43 biological processes, 39 molecular functions and 2 cellular components. The enriched pathways associated with annotated loci in the region are presented in **Supplementary Figure 10**. Within the genomic region for *qDTH5.1*, genes predicted to be associated with DTH include LOC_Os05g41450 (*OsDrAp2*), LOC_Os05g44180 (*FTL10*) and LOC_Os05g41070 (*BZIP42*) (**Table 6**; **Supplementary Figure 10**).

## 4 Discussion

The increasing global incidence of bacterial panicle blight (BPB), caused by *Burkholderia glumae*, continues to pose a serious threat to rice production worldwide (Tsushima et al., 1996; Nandakumar et al., 2007; Wang et al., 1991; Kim et al., 2010; Quesada-Gonzalez and Garcia-Santamaria, 2014; Riera-Ruiz et al., 2014; Zhou, 2014; Mondal et al., 2015), with notable expansion in the United States (Shew et al., 2019). The absence of reliable resistant cultivars or effective chemical control strategies underscores the urgency of identifying genetic determinants of resistance and elucidating their underlying mechanisms (Zhou, 2019). In this context, the moderately resistant cultivar Jupiter represents a valuable genetic resource for uncovering components of BPB resistance.

Our comparative transcriptomic analysis revealed a substantially stronger and more dynamic early defense response in Jupiter compared to the susceptible cultivar Bengal. At 6 hpi, Jupiter exhibited 2, 776 DEGs, compared to 1, 580 in Bengal, indicating a broader activation of defense-related pathways. The early transcriptional landscape in Jupiter was dominated by genes associated with cell wall modification, heat stress response, salicylic acid metabolism, pathogenesis-related (PR) proteins, redox processes, receptor kinases, and transcriptional regulation, particularly by WRKY transcription factors. This extensive early activation supports the concept that rapid and coordinated defense responses are central to effective resistance.

A key feature distinguishing Jupiter was the sustained expression of glutathione-S-transferases (GSTs) up to 24 hpi, unlike in Bengal. GSTs play critical roles in detoxification, conjugating glutathione to toxic compounds and modulating defense responses (Gullner et al., 2018). They are also involved in secondary metabolism and defense-related compound regulation (Zhang et al., 2024), and their accumulation has been linked to altered disease outcomes (Atkinson and Urwin, 2012). The prolonged GST expression in Jupiter probably contributes to enhanced detoxification capacity and cellular protection during infection.

Cell wall-associated defense mechanisms are considered as another important component of resistance. Plants rely on cell wall-embedded sensing systems to perceive stress signals (Atkinson and Urwin, 2012; Bacete et al., 2018), and modification of cell wall architecture is a well-established determinant of resistance (Bellincampi et al., 2014). Jupiter displayed a greater number of DEGs related to cell wall processes at 6 hpi, including the downregulation of pectin methyltransferases (PMTs), pectate lyases, and polygalacturonases. PMTs regulate apoplastic Ca^2+^ dynamics and influence cell wall plasticity (Wu et al., 2018), while their downregulation contributes to maintaining structural integrity under pathogen attack (Lionetti et al., 2007). Furthermore, PMTs modulate pectin methyl esterification, thereby affecting receptor accessibility and immune signaling (Wormit and Usadel, 2018). These observations suggest that Jupiter reinforces cell wall stability at an early infection stage, limiting pathogen ingress.

Transcriptional regulation also played a central role in the differential responses observed. MYB transcription factors were more highly expressed in Jupiter at 6 hpi, consistent with their roles in regulating defense genes, antimicrobial compound synthesis, and lignin biosynthesis (Li et al., 2019; Kajla et al., 2023; Falak et al., 2021). Their reduced expression at 24 hpi in both cultivars suggests that MYB-mediated regulation is primarily important during early infection. MYB factors also contribute to broader physiological processes, including hormone signaling and abiotic stress responses, indicating their role in integrating multiple stress signals (Katiryar et al., 2012).

Signal transduction pathways were also more active in Jupiter during early infection. Genes encoding S-locus glycoproteins, leucine-rich repeat (LRR) proteins, and calcium-dependent protein kinases (CDPKs) were differentially expressed, with greater activity in Jupiter at 6 hpi. Calcium signaling is a critical early event in plant defense, with rapid cytosolic Ca^2+^ fluctuations triggering downstream responses (Defalco et al., 2010; Zhang et al., 2014; Aldon et al., 2018). The enhanced expression of CDPK-related genes in Jupiter suggests a more calcium-dependent responsive signaling network. Additionally, receptor kinase genes such as LOC_Os04g58080 and LOC_Os01g52880 were more strongly upregulated in Jupiter, reinforcing the importance of early pathogen perception. The transient nature of these responses, particularly their decline by 24 hpi, further supports the role of their early and rapid activation in resistance.

Pathogenesis-related (PR) proteins were induced in both cultivars, indicating that pathogen recognition mechanisms are largely conserved. However, certain PR genes, including LOC_Os07g44450 (RGA1) and LOC_Os11g30210 (NBS-LRR protein), were more strongly expressed in Jupiter. Additional genes such as LOC_Os03g63150 (*Bph14*), a major brown planthopper resistance gene (Du et al., 2009), and LOC_Os04g44470, an alpha-amylase/subtilisin inhibitor for defense of the seed against microorganisms and for abiotic response, were uniquely upregulated in Jupiter. Despite these differences, the overall similarity in PR protein profiles suggests that variation in resistance is not solely due to pathogen recognition but rather to downstream signaling and response efficiency.

Other transcription factor families, including WRKY and bZIP, further contributed to the regulatory network. WRKY transcription factors, such as LOC_Os09g25060 (*OsWRKY76*) and LOC_Os05g25770 (*OsWRKY45*), play complex roles in modulating salicylic acid (SA) and jasmonic acid (JA) signaling pathways (Jimmy and Babu, 2015; Huang et al., 2021). *OsWRKY76* functions as a negative regulator of defense (Yokotani et al., 2013), whereas *OsWRKY45* positively regulates SA-dependent immunity (Jimmy and Babu, 2015; Yokotani et al., 2013). The downregulation of specific WRKY transcription factors can alleviate repression of defense-related genes and enhance stress responses, consistent with their role as molecular switches that integrate phytohormone signaling and transcriptional networks to exert both positive and negative control over plant immunity and stress tolerance (Wang et al., 2025; Khoso et al., 2022).

Similarly, two bZIP transcription factors, LOC_Os06g45140 and LOC_Os01g11350, were identified as differentially expressed genes in Jupiter and were significantly downregulated at 6 hpi. bZIP transcription factors are key regulators of rice disease resistance, modulating diverse molecular and physiological processes associated with biotic and abiotic stress responses, including reactive oxygen species (ROS) metabolism, SA signaling, and downstream defense gene expression (Lijuan et al., 2024; Mapuranga, 2026). Notably, LOC_Os06g45140 has been reported as a negative regulator of cold and drought stress responses (Liu et al., 2012), and its downregulation in Jupiter may enhance stress-responsive pathways, thereby contributing to increased disease resistance. This pattern is consistent with the elevated expression of several stress- and abiotic-related genes observed at 6 hpi in Jupiter. However, further functional studies are needed to clarify the specific role of LOC_Os06g45140 in disease resistance.

Heat shock factors (HSFs) appeared to be important contributors to BPB resistance. Beyond their classical roles in heat stress, HSFs act as molecular chaperones that stabilize defense-related proteins (Sangster and Quietsch, 2005; Shang et al., 2006). They may serve as a critical link between plant defense and heat stress responses, defense mechanisms are often influenced by elevated temperatures (Wu et al., 2022). LOC_Os01g42190, encoding a DnaJ heat shock protein, was strongly upregulated in both Jupiter and Bengal at 6 hpi and has been associated with resistance to brown planthopper and drought tolerance (Naik et al., 2018). Another HSF gene, LOC_Os03g14180, identified as a DEG in Jupiter, is known to confer thermotolerance in rice anthers during anthesis (Liu et al., 2020). Given that *B. glumae* infection occurs at this stage, its chaperone activity may contribute to resistance by stabilizing defense-related proteins. Additionally, LOC_Os01g08860, encoding the small heat shock protein OsHSP18.0-CII, functions as a molecular chaperone involved in both abiotic and biotic stress responses, including heat, salinity, and SA-mediated signaling (Kuang et al., 2017; Kaur et al., 2015; Chang et al., 2007). Its broad inducibility and demonstrated role in enhancing resistance to *Xanthomonas oryzae* pv. *oryzae* through SA-dependent pathways further support its protective function (Kuang et al., 2017). In Jupiter, the sustained upregulation of HSFs and associated chaperone activity up to 24 hpi likely contributes to maintaining protein homeostasis and stabilizing defense proteins, thereby supporting effective resistance, although prolonged activity may also influence host-pathogen interactions.

In addition to transcriptomic analyses aimed at elucidating the early-stage defense mechanisms of Jupiter during infection, we investigated quantitative trait loci (QTLs) associated with BPB resistance using a recombinant inbred line (RIL) population derived from a Bengal × Jupiter cross. To date, only a limited number of studies have reported QTLs for BPB resistance. Pinson et al. (2010) identified 12 QTLs using a RIL population derived from Lemont and TeQing (Pinson et al., 2010). Subsequently, Mizobuchi et al. (2013) mapped a major QTL on chromosome 1 conferring BPB resistance from the indica variety Kele (Mizobuchi et al., 2013). More recently, our group identified *qBPB3.1* as a major QTL associated with BPB resistance using a Trenasse × Jupiter RIL population (Ontoy et al., 2023). This locus overlapped with QTLs for sheath blight and days to heading, and also co-localized with a BPB QTL reported by Pinson et al. (2010) (Ontoy et al., 2023). Notably, *qBPB3.1* is also tightly linked to days-to-heading (DTH), potentially confounding its effect on BPB resistance (Ontoy et al., 2023).In the present study, we utilized another RIL population using Jupiter as the donor and Bengal as the susceptible parent to minimize this limitation and reduce linkage drag, as Bengal is genetically similar to Jupiter and exhibits a comparable DTH phenotype. This design allowed for the identification of genomic regions associated with BPB resistance with minimal interference from DTH trait.

Using this Bengal × Jupiter RIL population, we identified thirteen QTLs associated with BPB resistance. A stable QTL, *qBPB3.2*, located on the upper arm of chromosome 3, was consistently detected across field evaluations conducted in 2019, 2020, 2021, and 2023. This locus is adjacent to QTLs previously reported by Pinson et al. (2010) and Ontoy et al. (2023). Functional annotation of genes within this region revealed diverse biological roles. Notably, *qBPB3.2* was not linked to DTH, in contrast to *qBPB3.1* (Ontoy et al., 2023). Among the candidate genes identified, LOC_Os03g11160, LOC_Os03g11170, LOC_Os03g11180, and LOC_Os03g10750 were predicted to be associated with defense responses and stress-related functions. The first three genes encode cysteine proteinase inhibitors, class that regulate programmed cell death and defense signaling by inhibiting protease activity, thereby enhancing resistance to pathogens (Solomon et al., 1999). These inhibitors play a crucial role in suppressing pathogen-derived proteases, protecting immune-related proteins, and modulating defense pathways (Solomon et al., 1999; Paiva-Silva et al., 2025; Zhang et al., 2025). Additionally, LOC_Os03g10750 (*SPL35*) encodes a CUE domain–containing protein involved in ubiquitin-mediated signaling and vesicular trafficking pathways linked to plant immunity (Ma et al., 2019). Disruption of *SPL35* results in lesion mimic phenotypes characterized by hypersensitive response-like cell death, reduced chlorophyll content, increased accumulation of reactive oxygen species, particularly H_2_O_2_, and enhanced expression of defense-related genes, ultimately conferring resistance to both fungal and bacterial pathogens (Ma et al., 2019). These findings highlight *SPL35* as a key regulator of programmed cell death and immune signaling, linking ubiquitin-dependent processes to plant defense responses (Ma et al., 2019; Zhang et al., 2022).

Although several attractive candidate genes associated with BPB resistance were identified, including those encoding SPL35, cysteine proteinase inhibitors, and multiple heat shock factor (HSF)-related genes, the conclusions in this study are primarily based on transcriptomic profiling, QTL mapping, and correlative analyses. Therefore, functional validation of these candidate genes remains an important future direction. Follow-up studies using transient overexpression or gene silencing approaches, such as virus-induced gene silencing (VIGS), stable rice transformation, CRISPR/Cas9-mediated gene editing, or mutant phenotype analysis will be necessary to directly determine the contribution of these genes to BPB resistance and to further clarify the molecular mechanisms underlying the interaction between *Burkholderia glumae* and rice.

In addition to BPB resistance loci, we identified QTLs for DTH on chromosomes 2 and 5. The loci *qDTH2.1* and *qDTH2.2*, which largely overlap, contain candidate genes such as *OsLP2* and LOC_Os02g40100 that may play roles in flowering regulation, although their precise functions remain unclear and warrant further investigation through genetic and functional analyses. The *qDTH5.1* locus overlaps with a previously reported QTL identified in an F_2_ population derived from Jasmine 85 and Lemont (Pan et al., 1999). This genomic region contains key genes involved in flowering time regulation, including *OsFTL10*, *BZIP42* and *OsDrAp2*. *OsFTL10* acts as a florigen promoting flowering through the formation of the Flowering Activation Complex (FAC) under short-day conditions (Fang et al., 2019). In contrast, *BZIP42/HBF1* functions within the florigen repression complex (FRC), delaying flowering by regulating floral meristem identity (Li et al., 2023). Meanwhile, *OsDrAp2* (*OsHAPL1*) represses heading by interacting with DTH8 and Hd1 to suppress florigen gene expression via *Ehd1* (Zhu et al., 2017). Together, these genes illustrate the coordinated regulatory balance controlling flowering time in rice. In our previous with a RIL population derived from an early heading/BPB-susceptible variety ‘Trenasse’ and a late heading/BPB-resistant variety ‘Jupiter’ (Ontoy et al., 2023), BPB severity was strongly associated with the DTH trait, showing the co-localization of the major BPB QTL *qBPB3.1* with a major DTH locus *qHEAD3.1* on chromosome 3, suggesting potential pleiotropic or disease escape effects associated with flowering time. The parental lines in that population differed substantially in heading date, with Jupiter heading 8–12 days than Trenasse. In contrast, the RIL population used in the present study involved parents with relatively similar heading behavior (less than 2-day difference between Jupiter and Bengal) while maintaining contrasting BPB phenotypes. Under this reduced difference in DTH, *qBPB3.2* was still consistently detected across multi-year evaluations, suggesting that this locus likely contributes more directly to BPB resistance rather than being primarily confounded by heading-date effects.

The integration of transcriptomic and QTL analyses provided deeper insight into the genetic architecture of BPB resistance. Differentially expressed genes (DEGs) were widely distributed across the genome and did not overlap with candidate genes within major QTL region, *qBPB3.1* and *qBPB3.2*, although the genomic region spanning these two QTLs corresponds to previously described broad-spectrum quantitative disease resistance (BS-QDR) hotspots (Wisser et al., 2005). Notably, none of the candidate genes identified within the stable qBPB3.2 interval were detected as significantly differentially expressed genes (DEGs) in the RNA-seq analysis. This lack of co-localization suggests that the early transcriptional responses observed during BPB infection may primarily reflect upstream regulatory and signaling processes rather than direct expression of resistance-associated genes within the QTL region. Several factors may explain this observation, including tissue- or time-specific effects of the QTL that were not fully captured by the sampled infection stages, as well as resistance mechanisms mediated through constitutive expression, post-transcriptional regulation, epigenetic modification, protein-level activity, or coordinated pathway interactions rather than strong transcriptional induction. Moreover, QTL regions often contain complex gene clusters contributing to quantitative resistance through multilayered regulatory mechanisms (Wisser et al., 2005). Thus, the absence of overlap does not diminish the biological importance of *qBPB3.2*, but instead highlights the complex nature of BPB resistance, where stable QTL hotspots may function as integrative hubs of resistance while transcriptomic responses capture dynamic defense signaling across the genome. Integrative multi-omics approaches substantially enhance our understanding of complex traits associated with BPB resistance in rice. This comprehensive framework provides a deeper insight into the molecular mechanisms underlying plant–pathogen interactions and supports the development of targeted breeding strategies for improving rice disease resistance. Moreover, such integrative analyses provide a foundation for exploring the genetic architecture of other complex traits of quantitative disease resistance across crops, thereby accelerating the development of resilient, high-yielding varieties to address global food security challenges under changing environmental conditions.

## Data Availability

The GBS and RNA-seq datasets generated in this study are available in the NCBI database under BioProject accession number PRJNA1453607.
