# Supplementary material for: Characterization of resistance to bacterial panicle blight in rice revealed by transcriptome and QTL mapping analyses
Source: Front Plant Sci. 2026 Jun 3;17:1857873. doi: 10.3389/fpls.2026.1857873 (PMC13272157; doi:10.3389/fpls.2026.1857873)
Supplement: Supplementary file 1 [file Table1.docx]

Supplementary Material

# Supplementary Figures


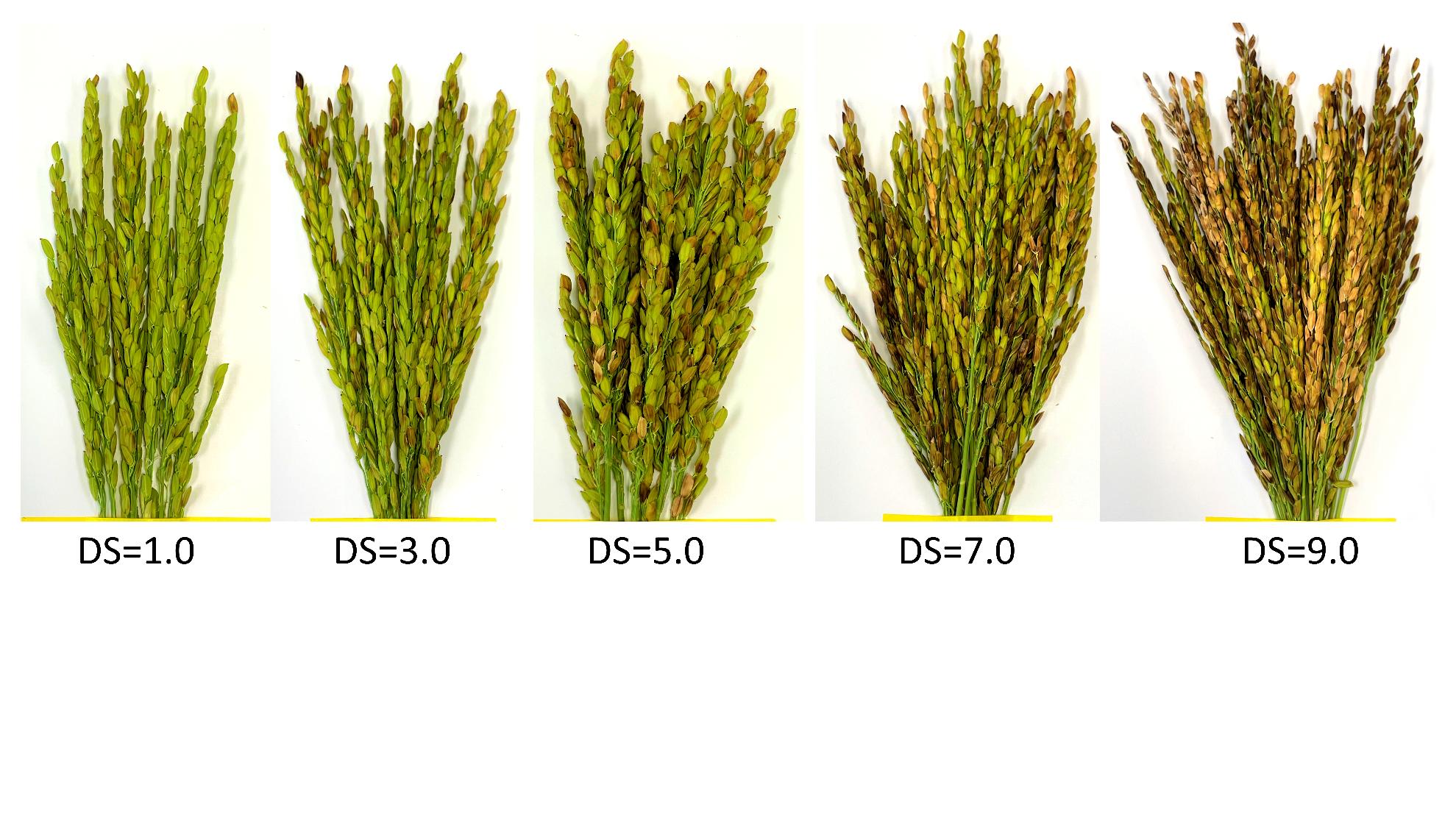


**Supplementary Figure 1.** The standard evaluation scoring for bacterial panicle blight in the field based on visual symptoms in the panicle.


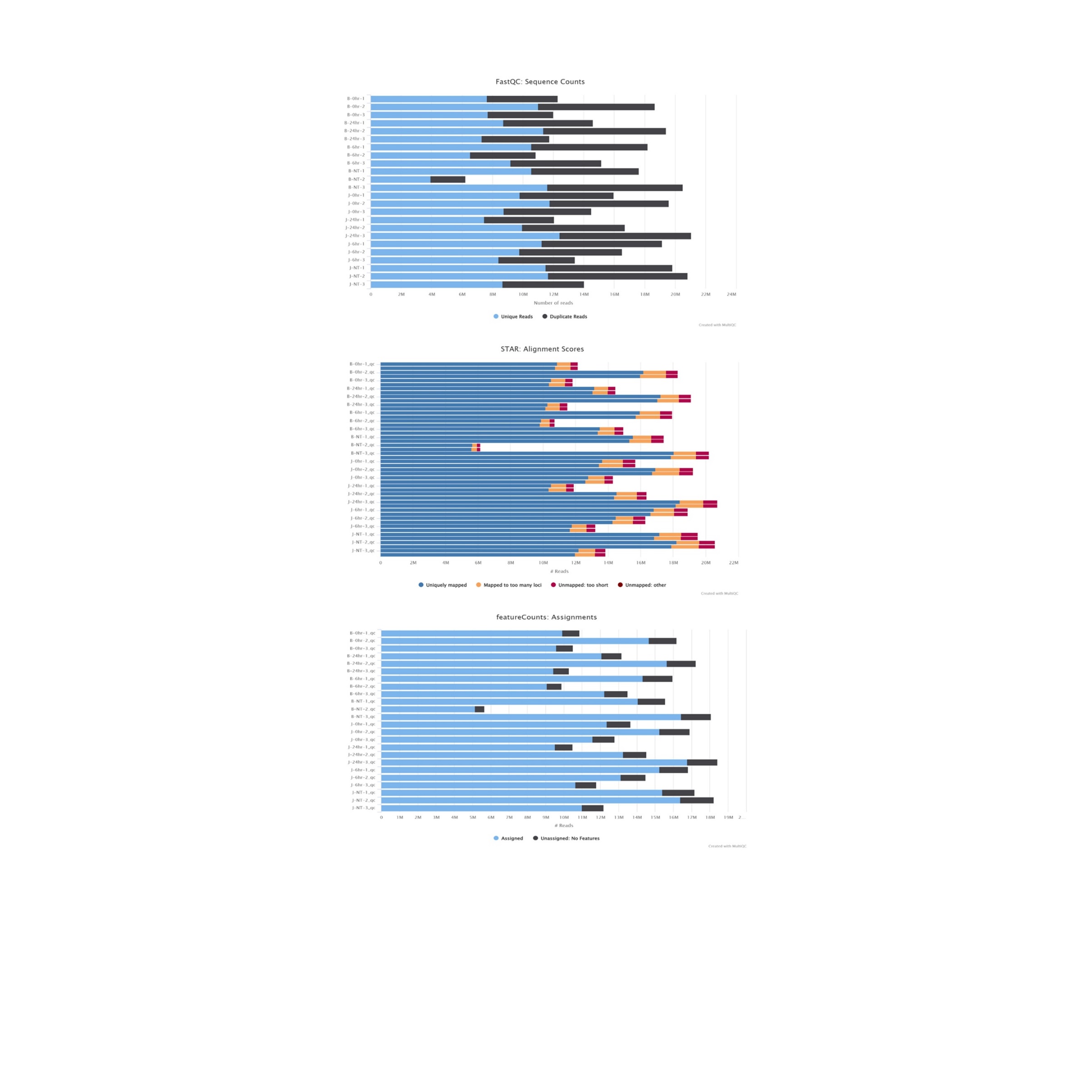


Supplementary Figure 2 (*Continued to the next page*).

(*Continued for Supplementary Fig. 2*)


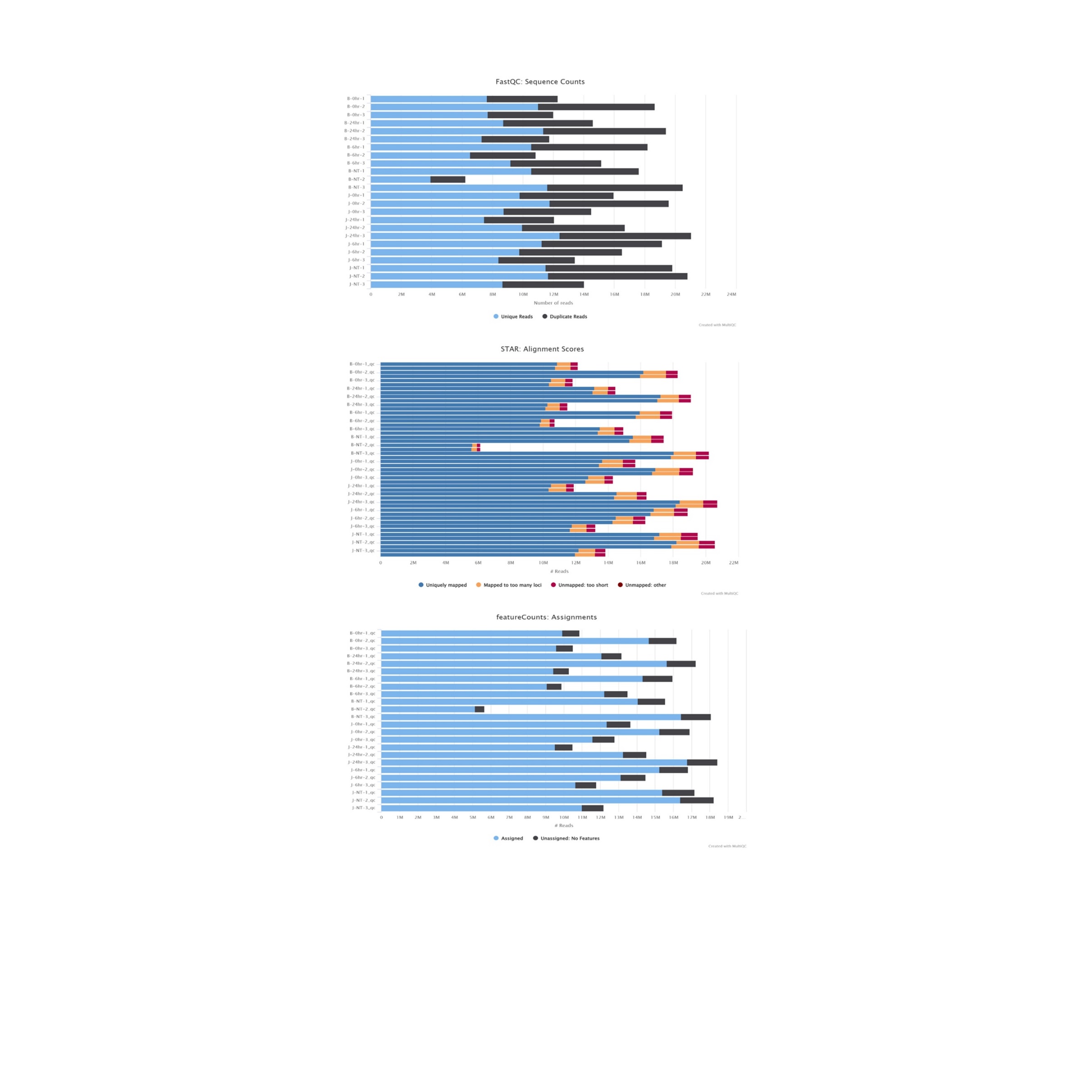


**Supplementary Figure 2.** Sequence counts for each sample and estimated duplicate read counts (upper panel), aligned reads for each sample generated by STAR version 2.7.10a (middle panel), and read counts mapped in exons for each sample using Subread featurecounts (lower panel).


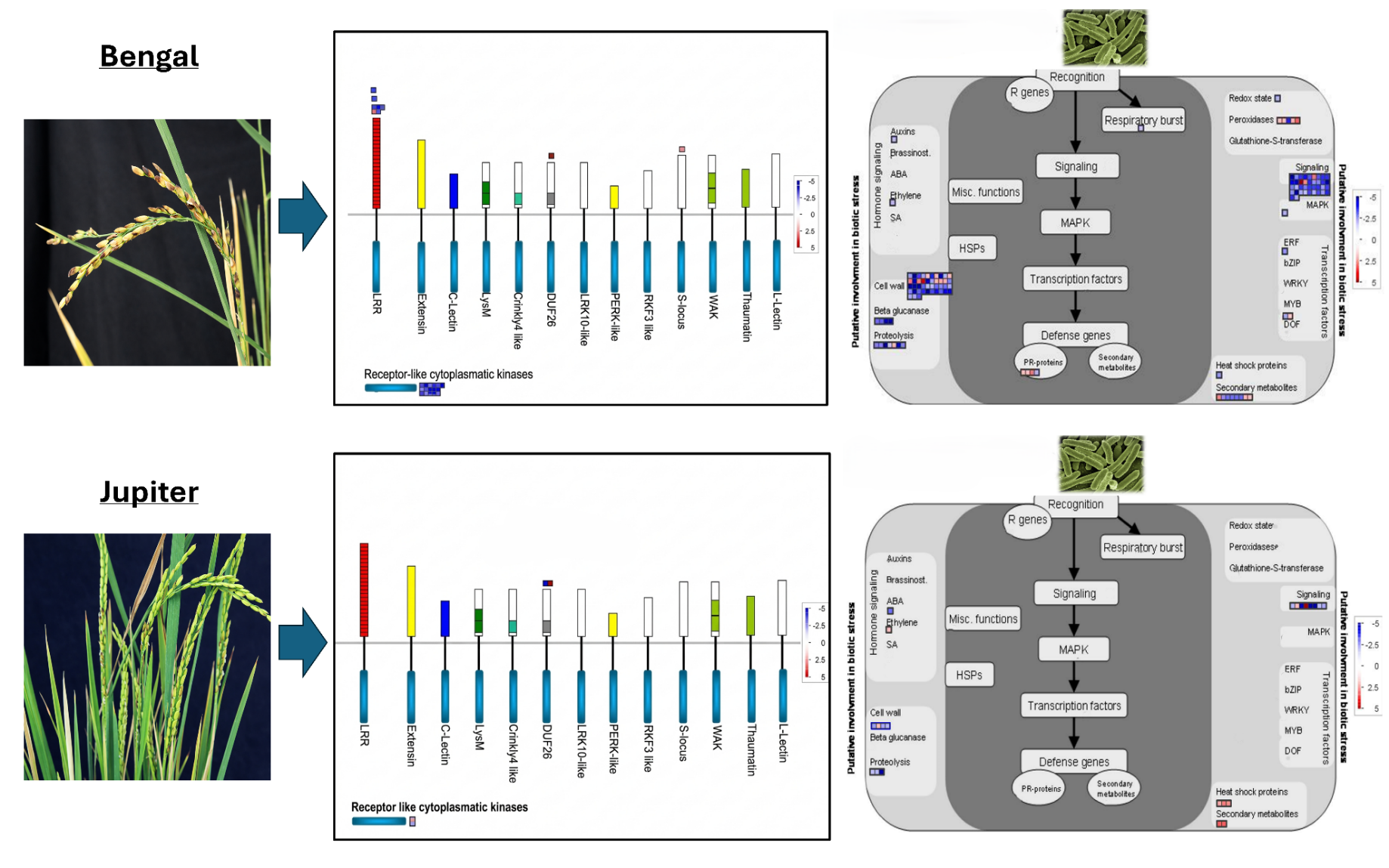


**Supplementary Figure 3.** Mapman analysis of significant DEGs (adjusted p-value ≤ 0.05 (FDR) and log2 fold change ≥ 2.0) involved in the biotic stress pathway in Jupiter and Bengal at 24 hpi of *B. glumae*. DEGs were binned to functional categories with log2 fold change values. Red represents upregulated loci while blue represents downregulated loci. ABA, abscisic acid; JA, jasmonic acid; SA, salicylic acid; PR, pathogenesis-related proteins; MYB, WRKY, ERF and bZIP, transcription factors; HSP, heat shock proteins/ factors.


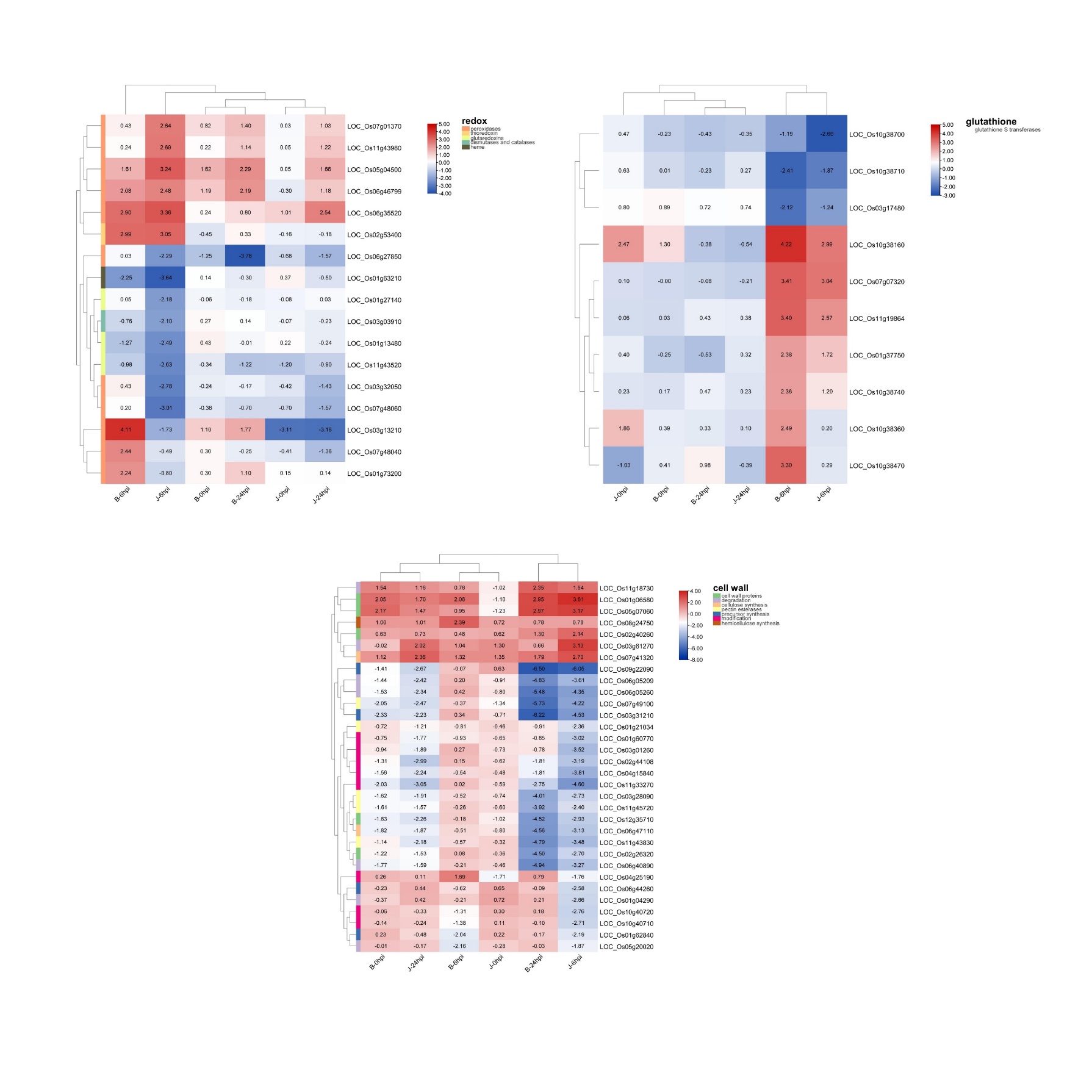


**Supplementary Figure 4.** Differentially expressed genes (FDR ≤ 0.05) associated with redox, glutathione metabolism, and cell wall between Jupiter and Bengal during the *Burkholderia glumae* infection. Blue indicates downregulation and red indicates upregulation.


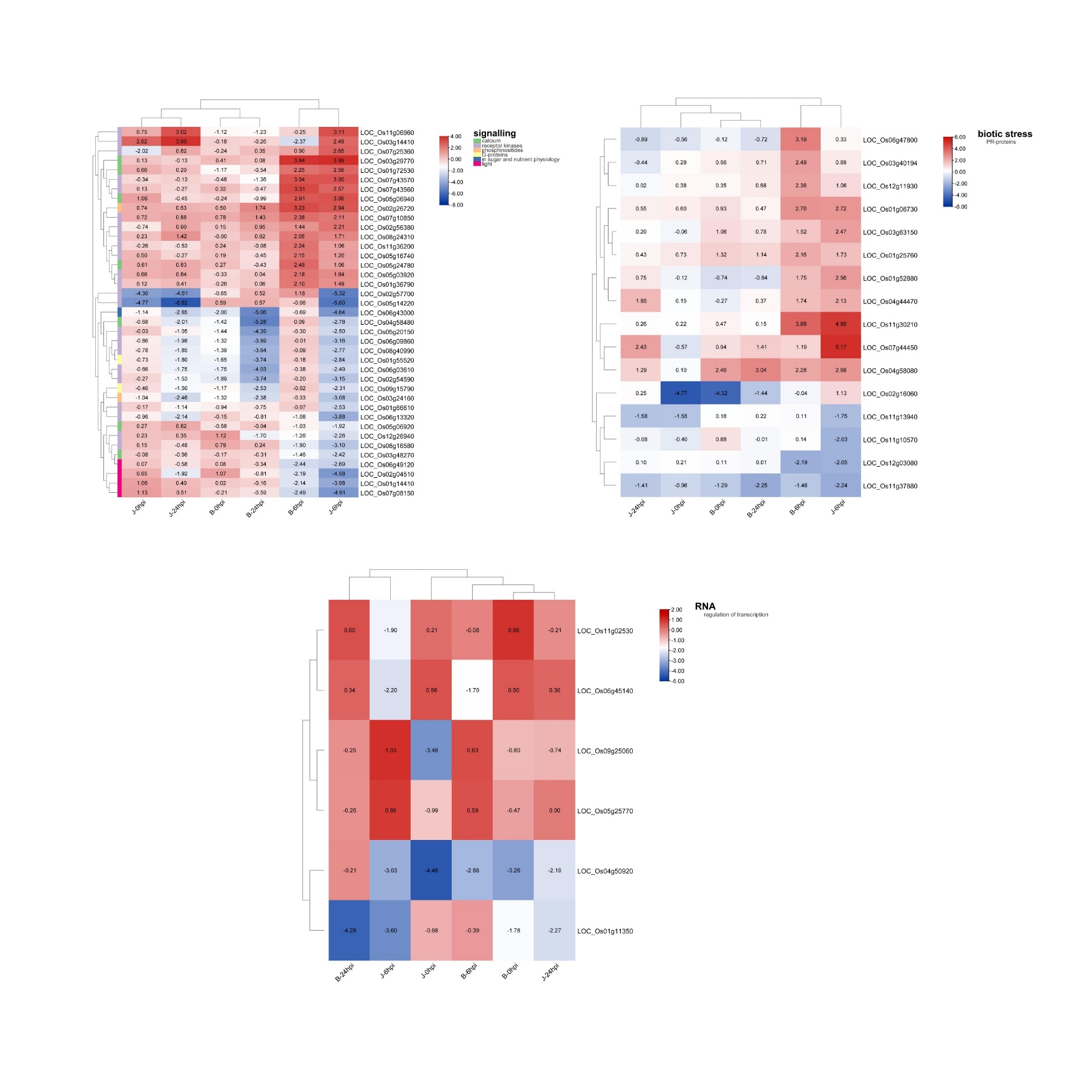


**Supplementary Figure 5.** Differentially expressed genes (FDR ≤ 0.05) associated with signaling, biotic stress, and RNA between Jupiter and Bengal across the *Burkholderia glumae* infection. Blue indicates downregulation and red indicates upregulation.


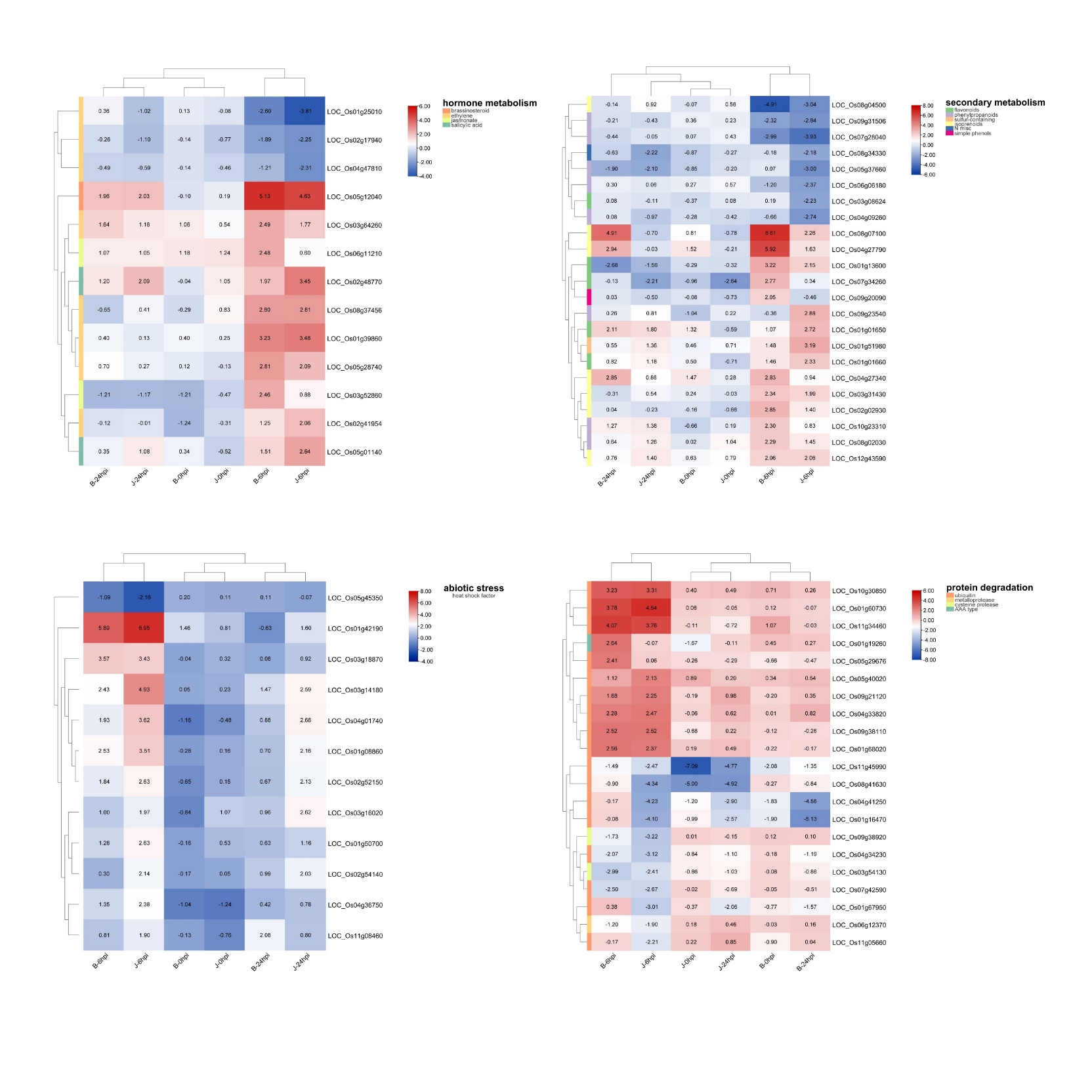


**Supplementary Figure 6.** Differentially expressed genes (FDR ≤ 0.05) associated with hormone metabolism, secondary metabolites, abiotic stress, and protein degradation between Jupiter and Bengal across the *Burkholderia glumae* infection. Blue indicates downregulation and red indicates upregulation.


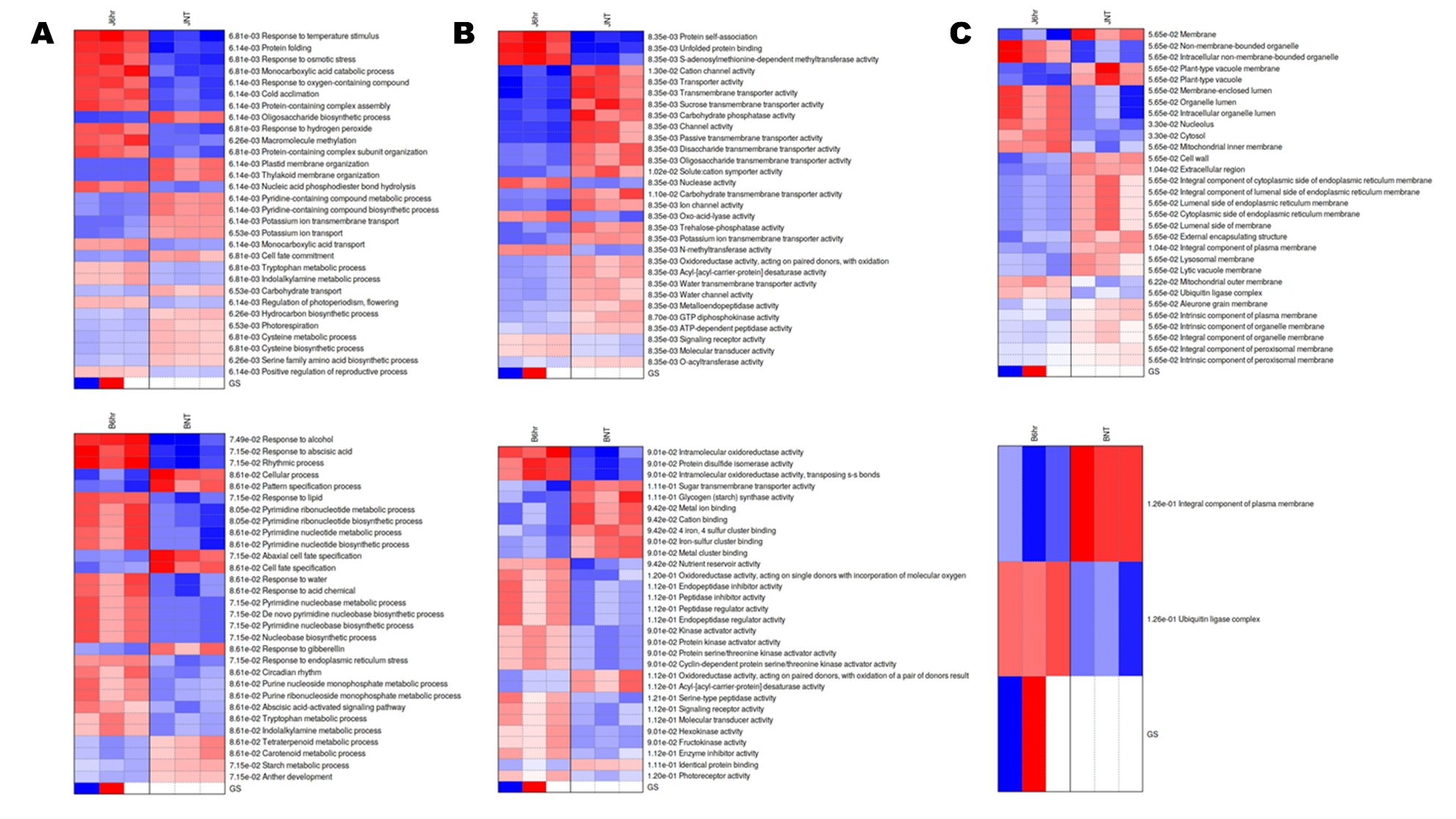


**Supplementary Figure 7.** Heatmap of predicted enriched pathways associated to DEGs that were identified in Jupiter and Bengal at 6 hpi of *B. glumae*. The red implies activated and blue for suppressed. (A) Biological processes (B) Molecular functions (C) Cellular components.


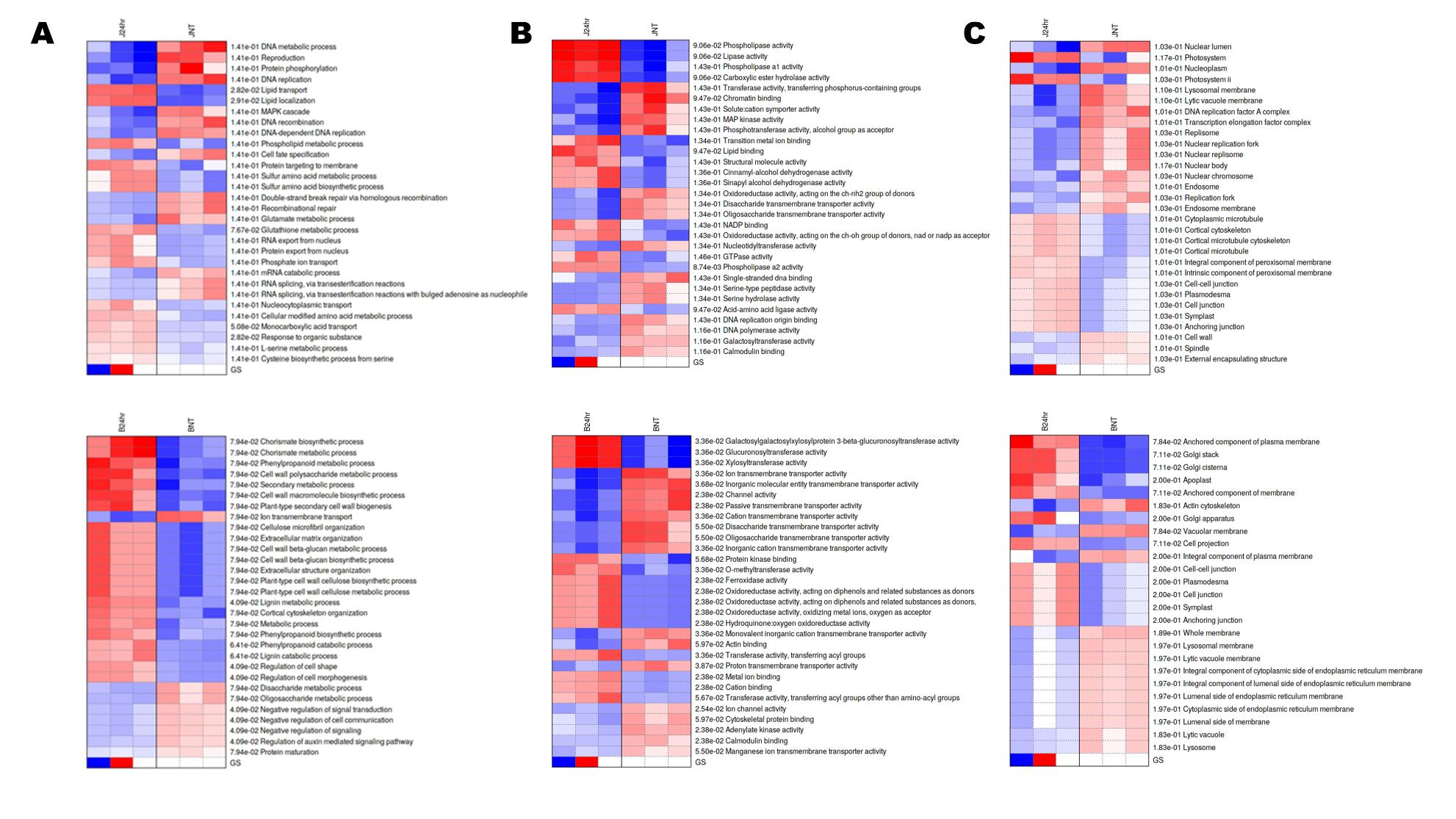


**Supplementary Figure 8.** Heatmap of predicted enriched pathways associated to DEGs (FDR ≤0.05) that were identified in Jupiter and Bengal at 24 hpi of *B. glumae*. The red implies activated and blue for suppressed. (A) Biological processes (B) Molecular functions (C) Cellular components.


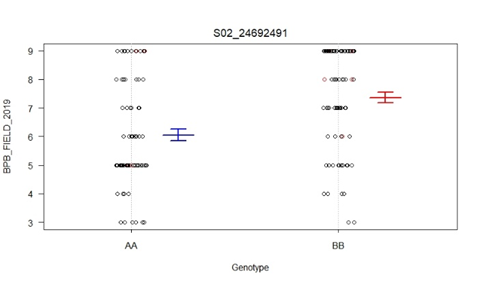

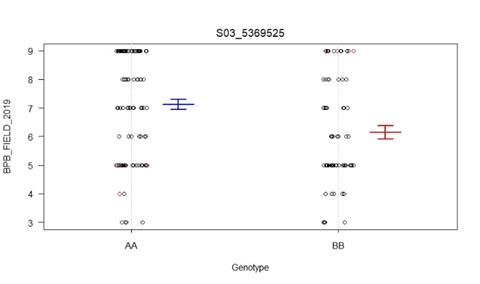


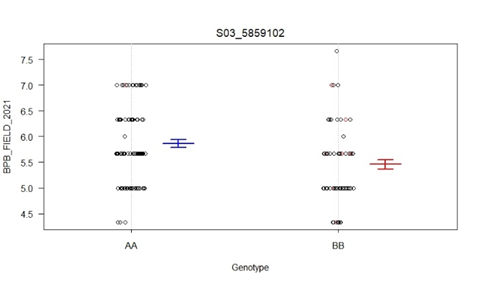

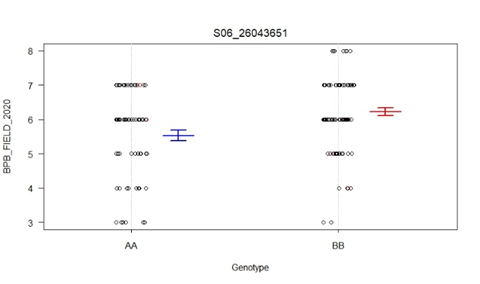


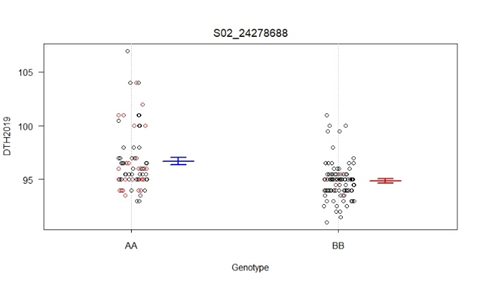

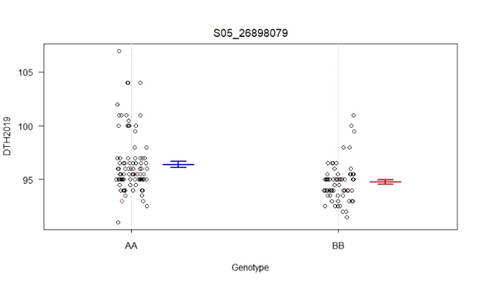


**Supplementary Figure 9.** The effect plot of markers associated with the significant positions for bacterial panicle blight (BPB) and days to heading (DTH) identified by non-parametric analysis (Kruskal-Wallis’ test). The AA and BB indicate Bengal and Jupiter genotypes, respectively.


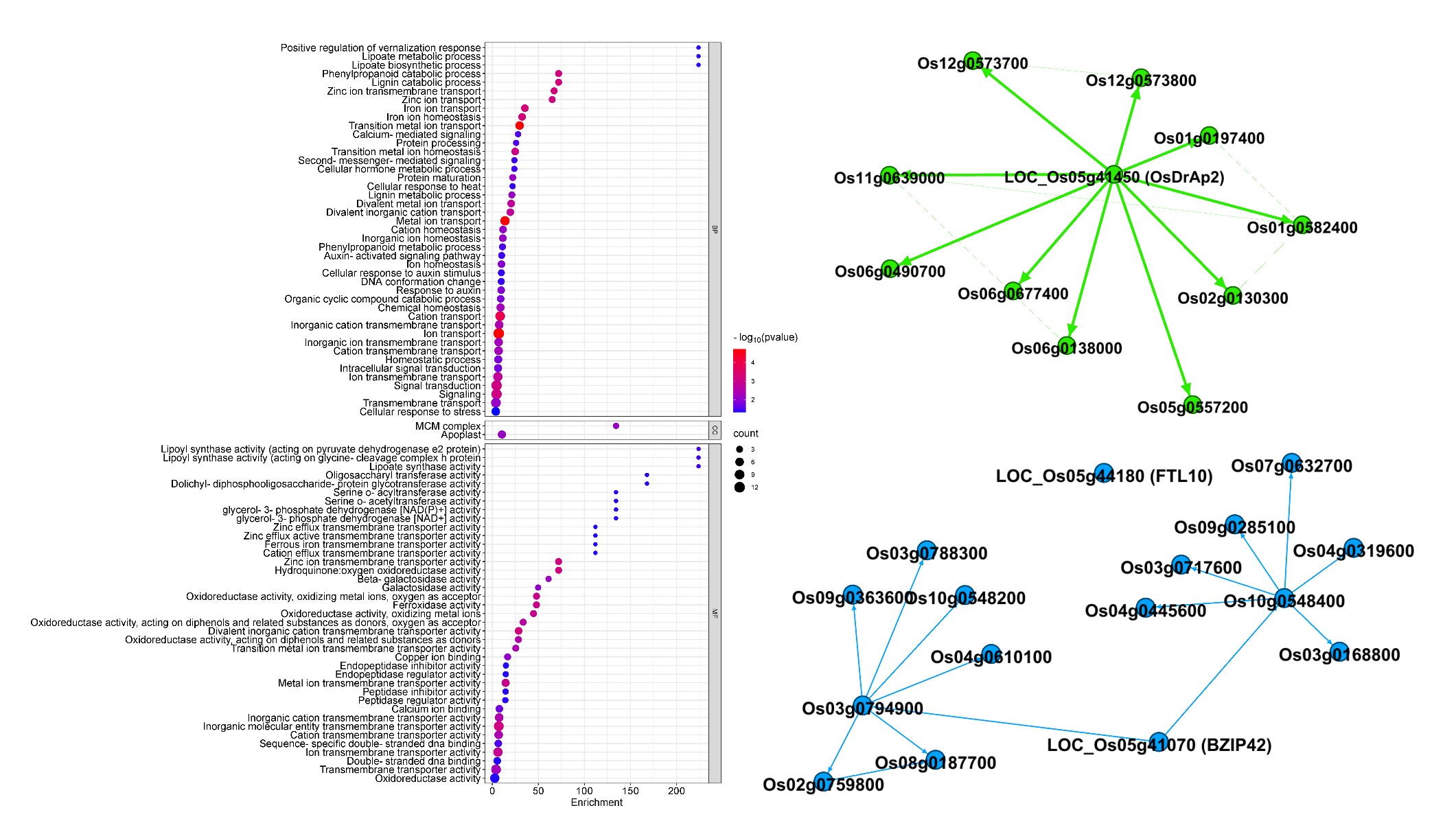


**Supplementary Figure 10.** Predicted enriched pathways (FDR ≤0.05) and networks associated to genes (*OsDrAp2*, *FTL10* and *BZIP42*) found to co-localized with *qDTH5.1* associated to days-to-50% heading (DTH) found in chromosome 5.

# Supplementary Tables

**Supplementary Table 1.** Candidate genes and associated primers for qPCR validation.

| **Candidate Genes** | **Type** | **Sequence** |
| --- | --- | --- |
| Assay Set (**LOC_OS07g44450**) | Forward | GAACCTTCGATCTCTCGATCAC |
|  | Reverse | GCTGAGGCCAAGCAGAATTA |
| Assay Set (**LOC_Os04g44470**) | Forward | GTGAAGAGGCTAGCAGGTATTG |
|  | Reverse | GCTCAGTGCTCACGAGTTATC |
| Assay Set (**LOC_Os09g25060**) | Forward | TGTCATCGGAGGGATTGTTG |
|  | Reverse | TGGTCACGCTTTGGACATAA |
| Assay Set (**LOC_Os06g45140**) | Forward | TCCAGCCAGCAGTTCAATAC |
|  | Reverse | GCTGAAGGAGAGTTTGGTTTCTA |
| Assay Set (**LOC_Os05g01140**) | Forward | CCACCTGCTCCACAATTACTAC |
|  | Reverse | GGAAGGAAGGATGGGATGAATG |
| Assay Set (**LOC_Os02g48770**) | Forward | TTCAACCTGCCGTTCTACTC |
|  | Reverse | GAGCTGGATGTGGCTTATGT |
| Assay Set (**LOC_Os03g14180**) | Forward | GAACGAGCGAATGAATGGAATAA |
|  | Reverse | GGAGAAACGCCAGAACTACA |
| Assay Set (**LOC_Os04g01740**) | Forward | CTCAAGGTCATCCGCAAGAA |
|  | Reverse | CCTCGTAGAACTTGGCGTAATC |
| Assay Set (**LOC_Os03g16020**) | Forward | ATCAAGGAGCAGGAGGAGAA |
|  | Reverse | CCATGGACGCCTTGATCTG |
| Assay Set (**LOC_Os01g08860**) | Forward | ATCGAAGCTTGAGTTGAGTGAG |
|  | Reverse | CACATCGCATACGGCATACA |
| Assay Set (**LOC_Os01g53220**) | Forward | CGTTCCCGTTCTCTGTTCTT |
|  | Reverse | CTAAACCGAGGCGAAGTTACA |

**Supplementary Table 2.** Test for normality of traits associated to BPB and DTH using Shapiro-Wilk.

| Traits | Normality Value | p-value |
| --- | --- | --- |
| BPB2019 FIELD | 0.88224 | 4.223e-10 |
| BPB2020 FIELD | 0.89916 | 3.983e-09 |
| BPB2021 FIELD | 0.91203 | 2.224e-08 |
| BPB2023 FIELD | 0.98202 | 0.03177 |
| DTH2019 | 0.85329 | 1.601e-11 |
| DTH2023 | 0.90038 | 4.321e-09 |

**Supplementary Table 3.** Predicted GO terms for biological process associated with *qBPB3.2* and their corresponding genes.

| Number of Genes | High level GO category | Genes |
| --- | --- | --- |
| 6 | Biological regulation | LOC_Os03g10650 LOC_Os03g11010 LOC_Os03g11040 LOC_Os03g11160 LOC_Os03g11170 LOC_Os03g11180 |
| 5 | Regulation of biological process | LOC_Os03g10650 LOC_Os03g11040 LOC_Os03g11160 LOC_Os03g11170 LOC_Os03g11180 |
| 5 | Regulation of metabolic process | LOC_Os03g10650 LOC_Os03g11040 LOC_Os03g11160 LOC_Os03g11170 LOC_Os03g11180 |
| 5 | Regulation of cellular process | LOC_Os03g10650 LOC_Os03g11040 LOC_Os03g11160 LOC_Os03g11170 LOC_Os03g11180 |
| 5 | Regulation of molecular function | LOC_Os03g10650 LOC_Os03g11040 LOC_Os03g11160 LOC_Os03g11170 LOC_Os03g11180 |
| 4 | Cellular process | LOC_Os03g10650 LOC_Os03g10950 LOC_Os03g11010 LOC_Os03g11040 |
| 3 | Response to defense response | LOC_Os03g11160 LOC_Os03g11170 LOC_Os03g11180 |
| 3 | Response to stress | LOC_Os03g11160 LOC_Os03g11170 LOC_Os03g11180 |
| 3 | Negative regulation of biological process | LOC_Os03g11160 LOC_Os03g11170 LOC_Os03g11180 |
| 2 | Cell cycle process | LOC_Os03g10650 LOC_Os03g11040 |
| 1 | Metabolic process | LOC_Os03g10950 |
| 1 | Localization | LOC_Os03g11010 |
| 1 | Nitrogen compound metabolic process | LOC_Os03g10950 |
| 1 | Cellular metabolic process | LOC_Os03g10950 |
| 1 | Primary metabolic process | LOC_Os03g10950 |
| 1 | Establishment of localization | LOC_Os03g11010 |
| 1 | Regulation of biological quality | LOC_Os03g11010 |
| 1 | Organic substance metabolic process | LOC_Os03g10950 |

**Supplementary Table 4.** Predicted GO terms for molecular function associated with *qBPB3.2* and their corresponding genes.

| Number of Genes | High level GO category | Genes |
| --- | --- | --- |
| 5 | Molecular function regulator | LOC_Os03g10650 LOC_Os03G11040 LOC_Os03g11160 LOC_Os03g11170 LOC_Os03g11180 |
| 5 | Enzyme regulator activity | LOC_Os03g10650 LOC_Os03G11040 LOC_Os03g11160 LOC_Os03g11170 LOC_Os03g11180 |
| 2 | Binding | LOC_Os03g10950 LOC_Os03g11370 |
| 1 | Catalytic activity | LOC_Os03g10950 |
| 1 | Transporter activity | LOC_Os03g11010 |
| 1 | Hydrolase activity | LOC_Os03g10950 |
| 1 | Transmembrane transporter activity | LOC_Os03g11010 |
| 1 | Ion binding | LOC_Os03g10950 |
| 1 | Organic cyclic compound binding | LOC_Os03g11370 |
| 1 | Heterocyclic compound binding | LOC_Os03g11370 |

**Supplementary Table 5.** Predicted GO terms for cellular component associated with *qBPB3.2* and their corresponding genes.

| Number of Genes | High level GO category | Genes |
| --- | --- | --- |
| 3 | Extracellular region | LOC_Os03g11160 LOC_Os03g11170 LOC_Os03g11180 |
| 3 | Membrane | LOC_Os03g10680 LOC_Os03g10870 LOC_Os03g11010 |
| 3 | Organelle | LOC_Os03g10650 LOC_Os03g11040 LOC_Os03g11370 |
| 3 | Intracellular | LOC_Os03g10650 LOC_Os03g11040 LOC_Os03g11370 |
| 3 | Intrinsic component of membrane | LOC_Os03g10680 LOC_Os03g10870 LOC_Os03g11010 |
| 3 | Membrane-bounded organelle | LOC_Os03g10650 LOC_Os03g11040 LOC_Os03g11370 |
| 3 | Intracellular organelle | LOC_Os03g10650 LOC_Os03g11040 LOC_Os03g11370 |
| 2 | Protein-containing complex | LOC_Os03g10650 LOC_Os03g11040 |
| 2 | Host cellular component | LOC_Os03g10650 LOC_Os03g11040 |
| 2 | Host cell part | LOC_Os03g10650 LOC_Os03g11040 |
| 1 | Plasma membrane | LOC_Os03g10870 |
| 1 | Cell periphery | LOC_Os03g10870 |

**Supplementary Table 6.** Predicted GO terms for biological process associated with *qDTH2.1* and *qDTH2.2* and their corresponding genes.

| Number of Genes | High level GO category | Genes |
| --- | --- | --- |
| 2 | modulation of process of another organism | LOC_Os02g40340 LOC_Os02g40400 |
| 3 | negative regulation of gene expression | LOC_Os02g40280 LOC_Os02g40340 LOC_Os02g40400 |
| 2 | negative regulation of translation | LOC_Os02g40340 LOC_Os02g40400 |
| 2 | negative regulation of cellular amide metabolic process | LOC_Os02g40340 LOC_Os02g40400 |
| 2 | negative regulation of cellular macromolecule biosynthetic process | LOC_Os02g40340 LOC_Os02g40400 |
| 3 | negative regulation of metabolic process | LOC_Os02g40280 LOC_Os02g40340 LOC_Os02g40400 |
| 3 | negative regulation of macromolecule metabolic process | LOC_Os02g40280 LOC_Os02g40340 LOC_Os02g40400 |
| 2 | regulation of translation | LOC_Os02g40340 LOC_Os02g40400 |
| 2 | negative regulation of cellular protein metabolic process | LOC_Os02g40340 LOC_Os02g40400 |
| 2 | regulation of cellular amide metabolic process | LOC_Os02g40340 LOC_Os02g40400 |
| 2 | negative regulation of protein metabolic process | LOC_Os02g40340 LOC_Os02g40400 |
| 2 | regulation of cellular macromolecule biosynthetic process | LOC_Os02g40340 LOC_Os02g40400 |
| 2 | negative regulation of biosynthetic process | LOC_Os02g40340 LOC_Os02g40400 |
| 2 | negative regulation of macromolecule biosynthetic process | LOC_Os02g40340 LOC_Os02g40400 |
| 2 | negative regulation of cellular biosynthetic process | LOC_Os02g40340 LOC_Os02g40400 |
| 3 | negative regulation of biological process | LOC_Os02g40280 LOC_Os02g40340 LOC_Os02g40400 |
| 2 | post-transcriptional regulation of gene expression | LOC_Os02g40340 LOC_Os02g40400 |
| 2 | negative regulation of nitrogen compound metabolic process | LOC_Os02g40340 LOC_Os02g40400 |
| 2 | negative regulation of cellular metabolic process | LOC_Os02g40340 LOC_Os02g40400 |
| 2 | regulation of cellular protein metabolic process | LOC_Os02g40340 LOC_Os02g40400 |
| 2 | negative regulation of cellular process | LOC_Os02g40340 LOC_Os02g40400 |
| 2 | regulation of protein metabolic process | LOC_Os02g40340 LOC_Os02g40400 |

**Supplementary Table 7.** Predicted GO terms for molecular function associated with *qDTH2.1* and *qDTH2.2* and their corresponding genes.

| Number of Genes | High level GO category | Genes |
| --- | --- | --- |
| 2 | RNA glycosylase activity | LOC_Os02g40340 LOC_Os02g40400 |
| 2 | rRNA N-glycosylase activity | LOC_Os02g40340 LOC_Os02g40400 |
| 2 | toxin activity | LOC_Os02g40340 LOC_Os02g40400 |
| 2 | catalytic activity acting on a rRNA | LOC_Os02g40340 LOC_Os02g40400 |
| 2 | hydrolase activity hydrolyzing N-glycosyl compounds | LOC_Os02g40340 LOC_Os02g40400 |
| 2 | hydrolase activity acting on glycosyl bonds | LOC_Os02g40340 LOC_Os02g40400 |
| 2 | catalytic activity acting on RNA | LOC_Os02g40340 LOC_Os02g40400 |
